# From chaos to order: the life history of *Hannaea inaequidentata* (Lagerstedt) Genkal and Kharitonov (Bacillariophyta), from initial cells to vegetative cells

**DOI:** 10.3897/phytokeys.162.56136

**Published:** 2020-10-09

**Authors:** Bing Liu, David M. Williams

**Affiliations:** 1 College of Biology and Environmental Science, Jishou University, Jishou 416000, China Jishou University Jishou China; 2 Department of Life Sciences, the Natural History Museum, Cromwell Road, London SW7 5BD, UK Natural History Museum London United Kingdom

**Keywords:** Buttressed central area, initial cell, perizonium, pre-normal vegetative cell, uniparental initial valve

## Abstract

This study presents observations on three species of *Hannaea* and documents and illustrates the life history of *H. inaequidentata*. We have divided the life history of *H. inaequidentata* into the following four series of successive stages: auxospore, initial cell, pre-normal vegetative cell, and normal vegetative cell. The initial cell has a cylinder-like and a frequently twisted outline, a longitudinal perizonium wholly covering the valve surface, and a disc-shaped incunabular scale, but lacks any transverse perizonium bands. The pre-normal vegetative cell cannot form ribbon-like colonies, has a wide variety of irregular outlines and is composed of two cell types: one with its epivalve composed of either the initial epivalve or the initial hypovalve, its hypovalve being newly formed, the other with both its epivalve and hypovalve newly formed. The normal vegetative cell has a regular outline and exhibits a significant length reduction so that the largest valve is at least four times longer than the smallest. From initial cell to normal vegetative cell, the developmental sequence goes from ‘chaos to order’ as happens in many phenomena in the universe. The lack of transverse perizonium bands may be the cause of the initial ‘chaos’ process during its developing period from the initial cell to the normal vegetative cell. The development of frustule/valve shape, central area, sternum, virga, vimine, rimoportula and ocellulimbus etc. during the life circle is summarised. In the genus *Hannaea*, some taxa lack the strongly buttressed central area as in *H. inaequidentata*, which also has almost parallel valve margins.

## Introduction

The diatom genus *Hannaea* R.M. Patrick (in Patrick and Reimer 1966, p. 131) was first used to accommodate *Ceratoneis arcus* (Ehrenberg) Kützing (1844, p. 104) and Ceratoneis arcus var. amphioxys (Rabenhorst) R.M. Patrick (in Patrick and Reimer 1966, p. 133), the former being the type of the genus: *Hannaea arcus* (Ehrenberg) R.M. Patrick (in Patrick and Reimer 1966). The need for the new name *Hannaea* in place of *Ceratoneis* Ehrenberg has been fully explained in Medlin and Mann (2007) and Van de Vijver and Ector (2020). *Hannaea* has recently been characterised as having valves “asymmetrical to the apical axis, usually with a small, unornamented tumid area on one side of the center of the valve” (Liu et al. 2019). Since the first use of the name *Hannaea* (as opposed to the name *Ceratoneis*), a number of species (and supra-specific taxa) have been added to the genus (ca. 13 in all, excluding varieties etc., see Liu et al. 2019) that fall into roughly four groups: (1) valves having uniseriate striae and a single rimoportula; (2) valves having biseriate striae and a rimoportula at both apices; (3) valves with poorly developed asymmetry to the apical axis; and (4) valves with (almost) parallel margins. Liu et al. (2019) note that from this diversity “two distinct groups can be identified […]”: one with uniseriate striae and a single rimoportula, the other with biseriate striae and two rimoportulae, one at each pole. It is not yet clear if either of these “two distinct groups” are monophyletic, if they are each other’s closest relatives (hence the genus being monophyletic), or if one or another of these two sub-groups is related to species outside the genus – Liu et al. (2019) suggest that the problem is worthy of investigation.

This study offers a contribution to further that investigation. We primarily focus on specimens identified as *Hannaea inaequidentata* (Lagerstedt) Genkal and Kharitonov (2008), a species with valves having almost parallel margins rather than the usual arcuate shape associated with many species of *Hannaea*. In an effort to understand the morphology and to help resolve the general relationships of *Hannaea*, this paper presents details on the entire life cycle of *H. inaequidentata*.

At present, very little is known of ‘araphid’ diatom life cycles and their ontogeny (reviews in Kaczmarska et al. 2001 and Kaczmarska et al. 2013, see Jewson and Bixby 2016). Herein, we document the morphological changes observed in the transition from what has been termed post-auxospore cells to the ‘normal’ vegetative cells, noting the changes in particular features of the valve during development. For comparative purposes we include brief descriptions of specimens identified as Hannaea cf. arcus and Hannaea cf. baicalensis (*Hannaea baicalensis* Genkal, Popovskaya & Kulikovskiy, 2008). The latter is possibly a new species from Lake Baikal, Siberia (see Williams 2019); the identity of the former remains uncertain. Both are used here simply as examples of the variation in *Hannaea*.

## Material and methods

The diatom samples were collected from three different regions that are some distance from each other. The samples for Hannaea cf. arcus were collected from a tributary of the Datong River in Qinghai province of China in August 2018. The specific sampling site is in Bazha town, Huzhu County, Qinghai province, its coordinates are 37.03684°N and 102. 415849°E with an elevation of 2801 m a.s.l. Temperature, pH, and conductivity were measured in situ with a portable multimeter (HQ40D, HACH Company): pH = 8.92 ± 0.02, conductivity = 230.6 ± 0.1 μS/cm, temperature = 15.4 ± 0.1 °C.

The samples of *Hannaea inaequidentata* were collected from Heiwan River at the foot of Fanjing Mountain in Guizhou province of China in December 2015. The specific sampling site is beside Longquan Temple which is within the Fanjing Mountain National Nature Reserve, Jiangkou County, Guizhou province. Its coordinates are 27.860093°N and 108. 764229°E with an elevation of 532 m a.s.l. Temperature, pH, and conductivity were measured in situ with a portable multimeter (HQ40D, HACH Company): pH = 7.7 ± 0.1, conductivity = 49.7 ± 0.2 μS/cm, temperature = 9.4 ± 0.1 °C.

The samples for Hannaea cf. baicalensis were collected from Lake Baikal, Siberia, as part of a Darwin Initiative (DI) project (Flower and Williams 1999; see http://www.geog.ucl.ac.uk/ecrc/enclosed/dardata.htm). Duplicate materials for the DI Lake Baikal collections are located in CAS (California Academy of Science), E (Royal Botanical Gardens, Edinburgh), Minsk (Laboratory of Quaternary Geology, Minsk, Belarus) and the Limnological Institute, Irkutsk, Russia.

The samples from China were scraped from stone surfaces using toothbrushes, then washed into 100 ml sampling bottles and fixed with 70% ethanol. Permanent slide preparation, light microscopy observation, and scanning electron microscopy observation follow Liu et al. (2020). A similar protocol was used for the Baikal samples.

### Terminology and abbreviations

*Valve morphology*: We mostly follow Ross et al. (1979) and Cox and Ross (1981) for valve structure terminology and Williams (1985) for girdle band terminology. With respect to the valve central area (the “unornamented tumid area” of Liu et al. 2019, the “unilateral inflation” of Bixby et al. 2005, and other descriptions), which is of some significance for species in the genus *Hannaea*, we follow and comment upon Bixby et al. (2005).

*Life cycles*: We have mostly followed Kaczmarska et al. (2013, and, to a lesser extent, its precursor, Kaczmarska et al. 2001) for life cycle terminology. We introduce a few new terms that allow more precise documentation of the various stages observed in *Hannaea inaequidentata*. Below we refer to the vegetative stages during which the cells exhibit regular shapes as ‘normal’, hence ‘normal vegetative cells’. In this sense, certain ‘pre-normal cells’ can be identified.

Pre-normal vegetative period: The time between immediately after the initial cell’s first division and the presence of the first new normal vegetative cells. The cell, frustule, and valve occurring during this period can be termed ‘pre-normal vegetative cell, frustule, and valve’. Kaczmarska et al. stated that “It is often convenient to refer to the first few mitotic generations of cells produced by division of the initial cell as **post-initial cells**” (Kaczmarska et al. 2013, p. 266). Post-initial cells will include normal vegetative cells, so using the term ‘pre-normal vegetative period’ divides the life history into the following series of successive stages: auxospore, initial cell, pre-normal vegetative cell, and normal vegetative cell.

Uniparental initial valve period: The time between the first-generation valve from the initial cell and the termination of initial valves’ division. There are two types of frustule: one is composed of an initial epivalve and a non-initial hypovalve (the newly formed valve), the other is of one initial hypovalve (as epivalve in the first-generation frustule) and a non-initial hypovalve (the newly formed valve). Both the structure of the initial epivalve and initial hypovalve can be maintained for a few generations.

Standard abbreviations have been used throughout, e.g., LM = light microscopy; SEM = scanning electron microscopy, etc. Other abbreviations used in the text and figures are: Ev = epivalve; Hv = hypovalve; B1 = valvocopula; B2 = second band, copula; B3 = third band, copula; B4 = fourth band, copula; NB3 = new-born third band for hypovalve; NB4 = new-born fourth band for hypovalve.

Author names follow the International Plant Names Index (IPNI) (https://www.ipni.org/), herbarium names follow the Index Herbarium (http://sweetgum.nybg.org/science/ih/).

## Results

### Hannaea cf. arcus

Figs 1–7, 10–17, 18–21

**Figures 1–9.**
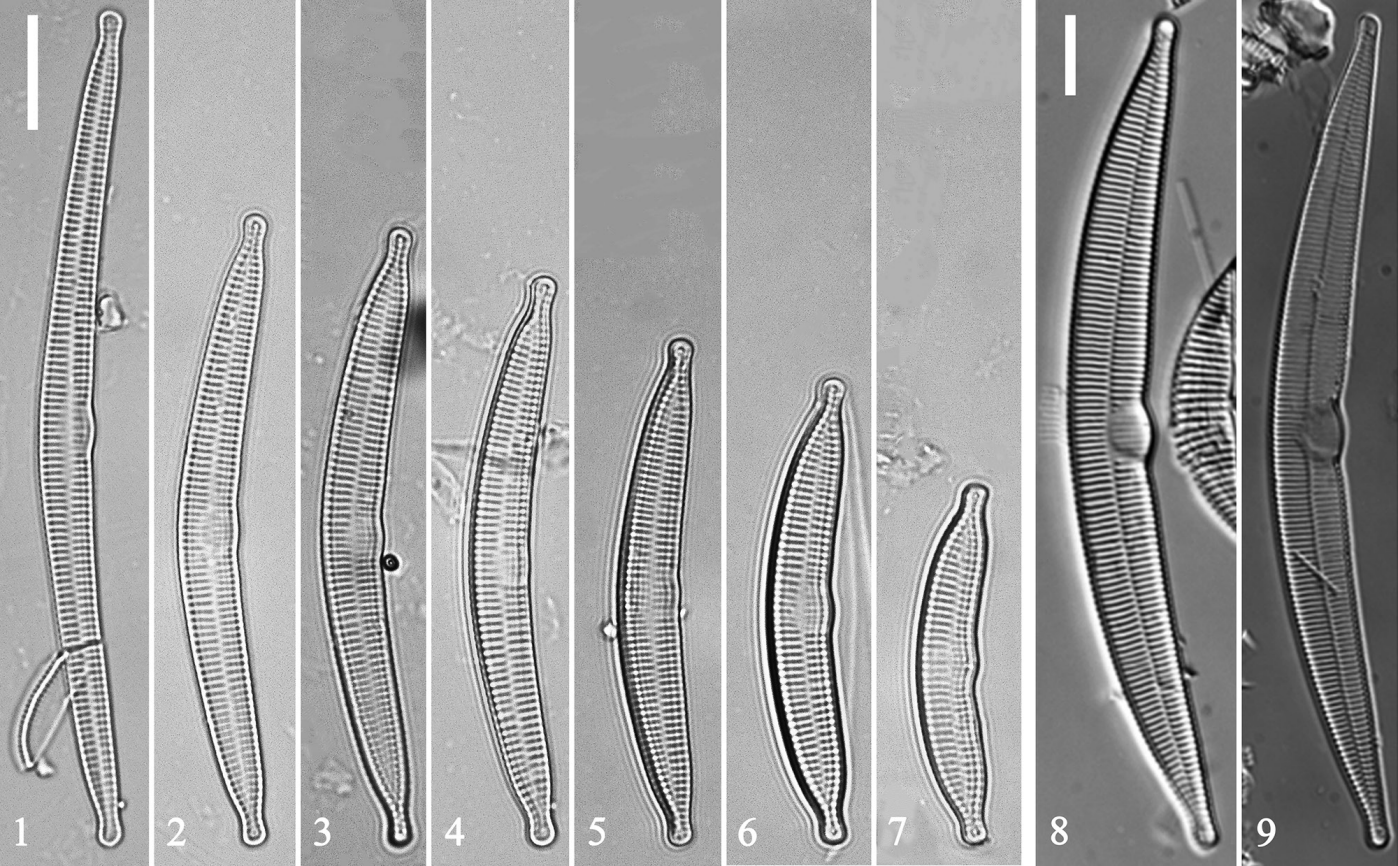
Hannaea cf. arcus and Hannaea cf. baicalensis, LM**1–7** seven valves showing valve size diminution series for Hannaea cf. arcus**8, 9** two valves of Hannaea cf. baicalensis. Scale bar: 10 μm (**1, 8**).

Observation: LM: Valves gently arcuate, with capitate apices (Figs 1–7). Valve dimensions (n = 31): length 31–85 μm, width 4.5–7.5 μm; striae almost uniform until reaching poles. Central area as swelling on ventral side, reaching sternum, with faint ghost striae. Sternum narrow, linear. Striae mostly alternate, parallel, stria density 15–17 in 10 μm.

**Figures 10–17.**
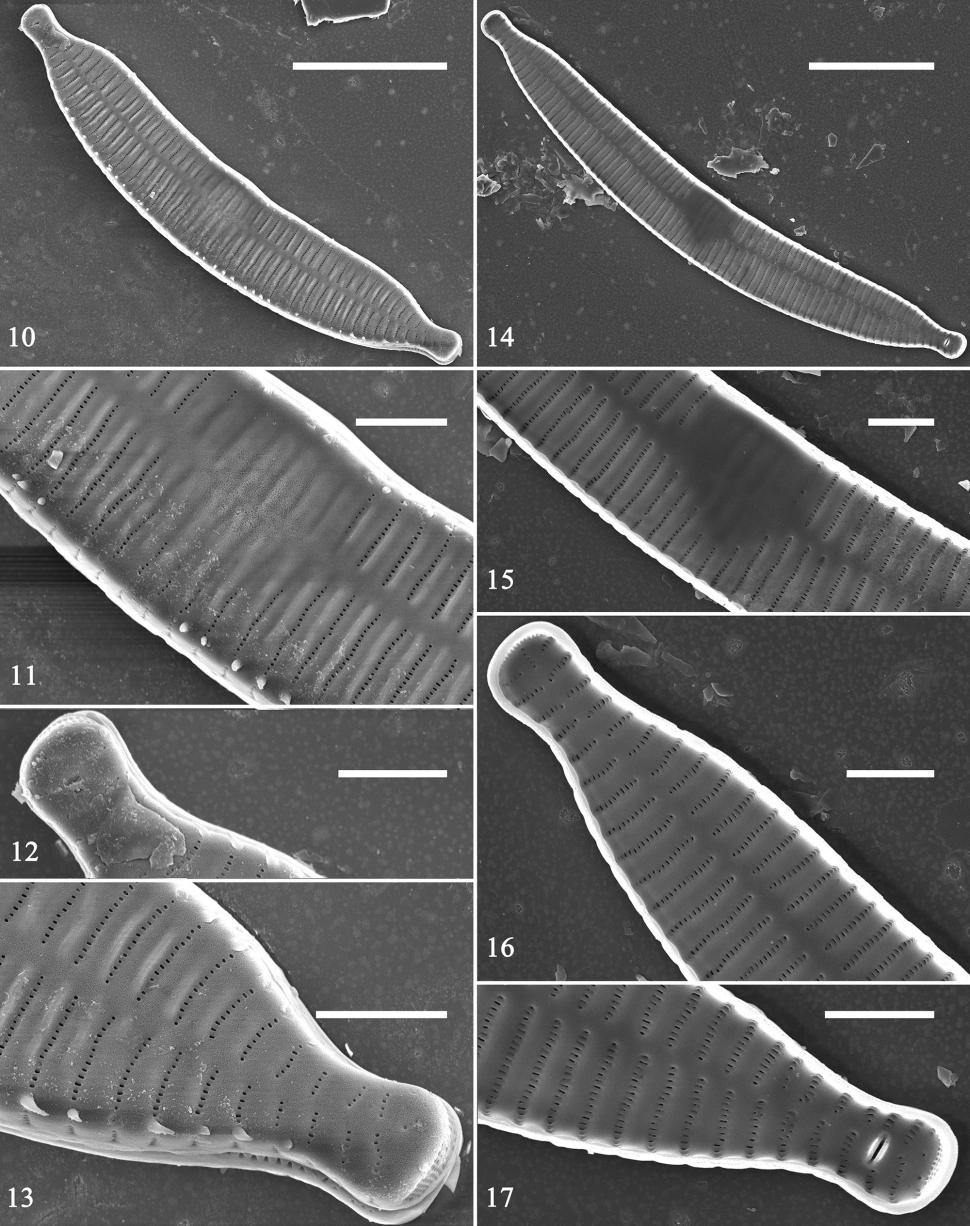
Hannaea cf. arcus, SEM**10–13** external view of valve, note linking spines and central area **14–17** internal view of valve, note central area lacking buttressing (**15**). Scale bars: 10 μm (**10, 14**), 2 μm (**11–13, 15–17**).

**Figures 18–21.**
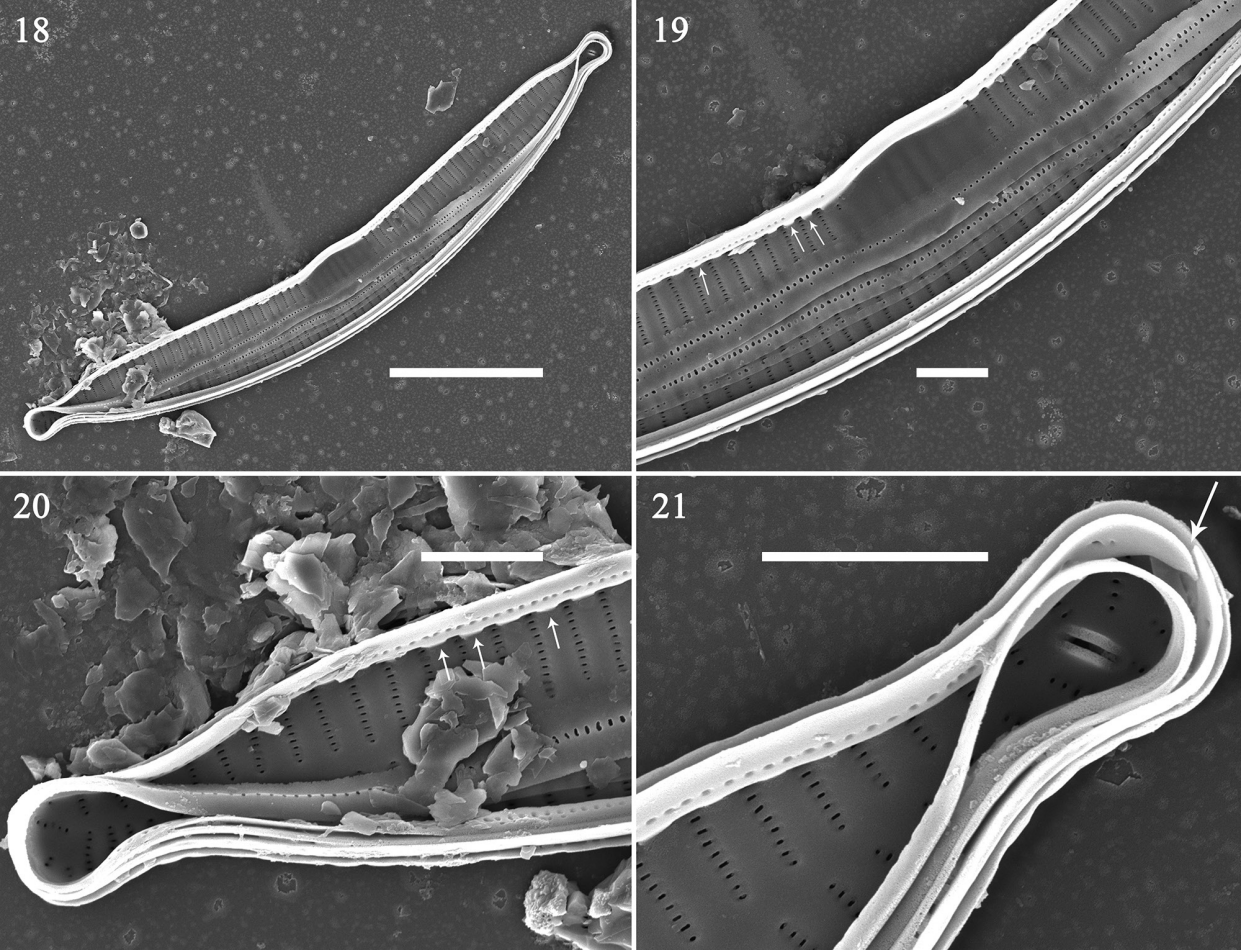
Hannaea cf. arcus, SEM**18** complete valve with valvocopula. **19–21** details of Fig. 18, note valvocopula with sawtooth-shaped projections attached to valve (**19, 20**, arrows), valvocopula open at one pole (**21**, arrow). Scale bars: 10 μm (**18**), 2 μm (**19–21**).

SEM: Virgae slightly raised, wide, vimines much smaller, evenly spaced (Figs 10–21); spines present, situated at valve margin, between two adjacent virgae, interrupting striae (Figs 10–13). Striae uniseriate, with round or oblong areolae. Central area tumid, with transversely raised virgae, faint ghost striae, lacking buttressing (Figs 14, 15). Sternum central, linear. One rimoportula per valve, with paired lips, situated at apex (Figs 16, 17); ocellulimbus located on valve margin at each pole, composed of vertical rows of ca. 5–10 poroids. Valvocopula open, same shape as valve, closely attached to mantle interior, surrounding valve margin (Figs 18, 21). Each valvocopula with single row of poroids bisecting pars interior and exterior, located at mid-line (Figs 19–21). Valvocopula with sawtooth-shaped projections attached to valve, internally visible over each virga (Figs 19, 20, arrows).

### Hannaea cf. baicalensis

Figs 8, 9, 22–34

Observation: LM: Valves arcuate, with capitate to sub-capitate apices (Figs 8, 9). Valve 40–150 μm long, 4–12 μm wide. Striae almost uniform until reaching poles. Central area as definite swelling on ventral side, reaching sternum, ghost striae just beyond. Sternum narrow, linear, well-defined. Striae mostly alternate, parallel, striae density 10–15 in 10 μm.

**Figures 22–28.**
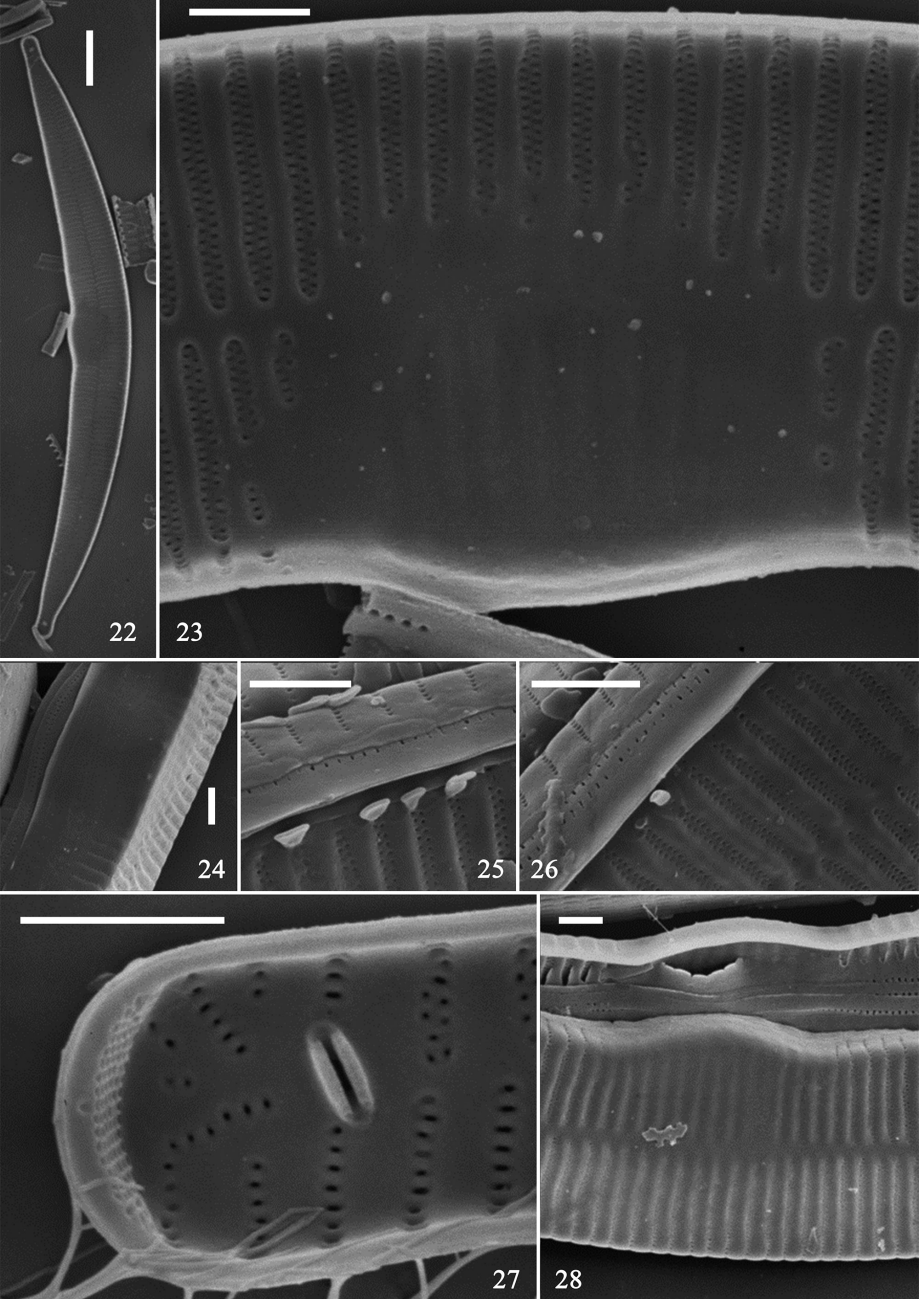
Hannaea cf. baicalensis, SEM. **22** complete valve, internal view, note rimoportula at each pole **23–28** detail of valve structure. Scale bars: 10 μm (**22**), 2 μm (**23–28**).

**Figures 29–34.**
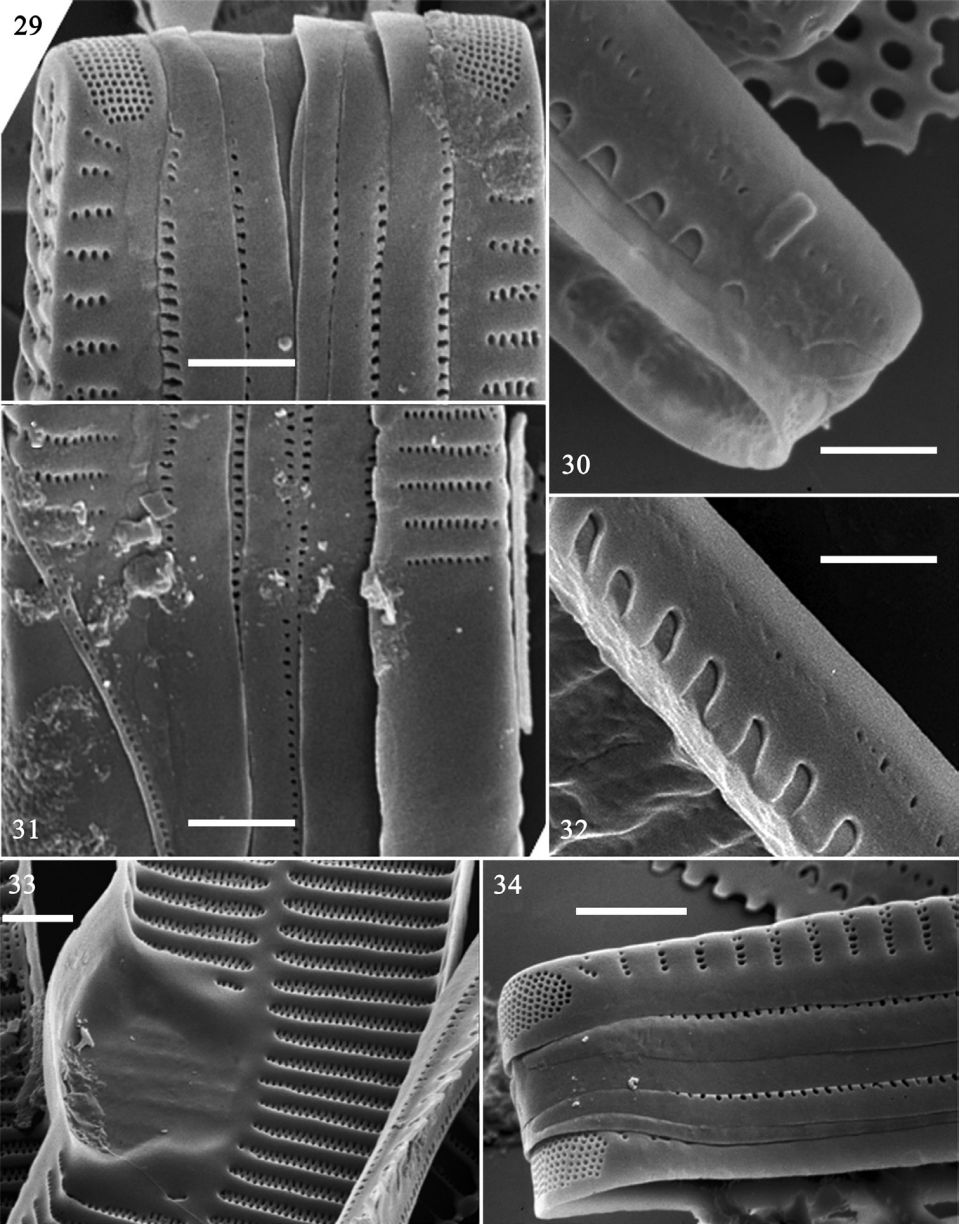
Hannaea cf. baicalensis, SEM. **29–32, 34** details of cingulum **33** central area of valve, internal view, note buttressed central area. Scale bar: 2 μm (**29–34**).

SEM: Virgae raised, relatively wide, vimines much smaller, sunken but evenly spaced (Figs 22–28); spines present as short ill-defined spurs, approaching spathulate, most emanating from vimines (Figs 24, 25, 28). Striae biseriate, with round areolae occurring opposite each other, uniseriate at poles, formed by merging vimines. Central area tumid, with transversely raised virgae, vimines filled in forming ghost striae, buttressing demarcating either side of central area, fusing with sternum (Figs 23, 33). Sternum central, linear, level with virgae. Rimoportula at each pole, two per valve, each with simple paired lips (Fig. 27); ocellulimbus well-developed, located on valve margin at each pole, composed of parallel vertical rows of ca. 15–20 poroids (Figs 29, 34). Cingulum composed of simple open bands, possibly four per valve, single row of poroids either side of pars media, only par exterior (usually) visible (Figs 29, 31, 34). Valvocopula with fringed edge to affix internal portion of valve (Figs 30, 32). All bands similar structure, lacking pleurae.

### *Hannaea inaequidentata*

Figs 35–136

Observation:

*Normal vegetative colony and frustule* Figs 35–47.

**Figures 35–37.**
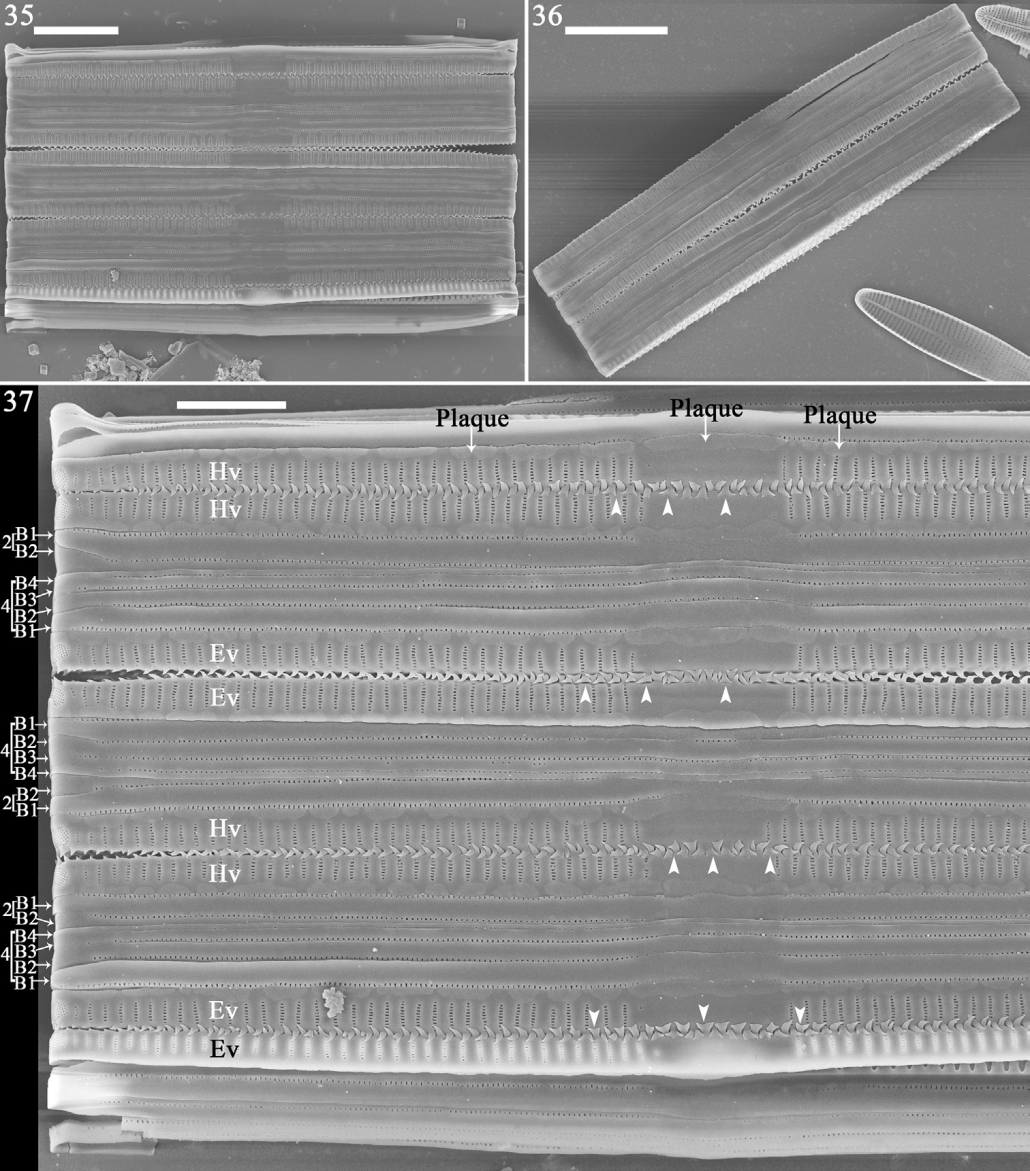
*Hannaea inaequidentata*, girdle view, SEM**35** colony with ca. 5 frustules **36** colony with two frustules **37** detail of Fig. 1, showing epivalves and hypovalves, distinct mantle plaques, fork-shaped interlocked linking spines at valve middle (arrowheads) and more acute spines towards each apex; note 4:2 configuration of girdle bands in three normal but not dividing vegetative frustules. Scale bars: 10 μm (**35, 36**), 5 μm (**37**).

**Figures 38, 39.**
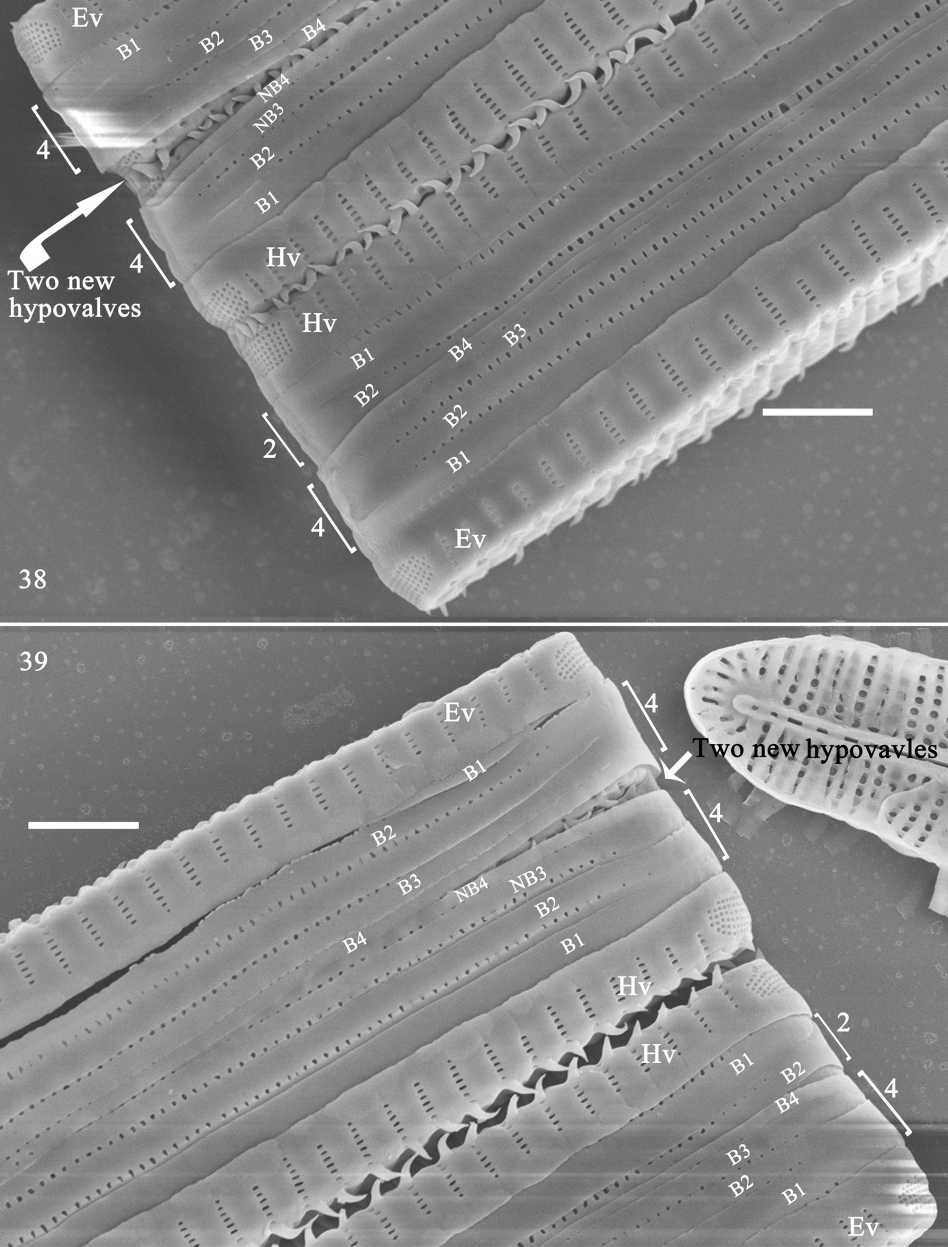
*Hannaea inaequidentata*, girdle view, SEM. **38, 39** details of two apices from Fig. 36, showing epivalves and hypovalves, 4:2 configuration of girdle bands in normal but not dividing vegetative frustule (lower frustule), 4:4 configuration of girdle bands in dividing vegetative frustule (upper frustule); note two new hypovalves (arrows) are interlocked by linking spines. Scale bars: 2 μm.

**Figures 40–47.**
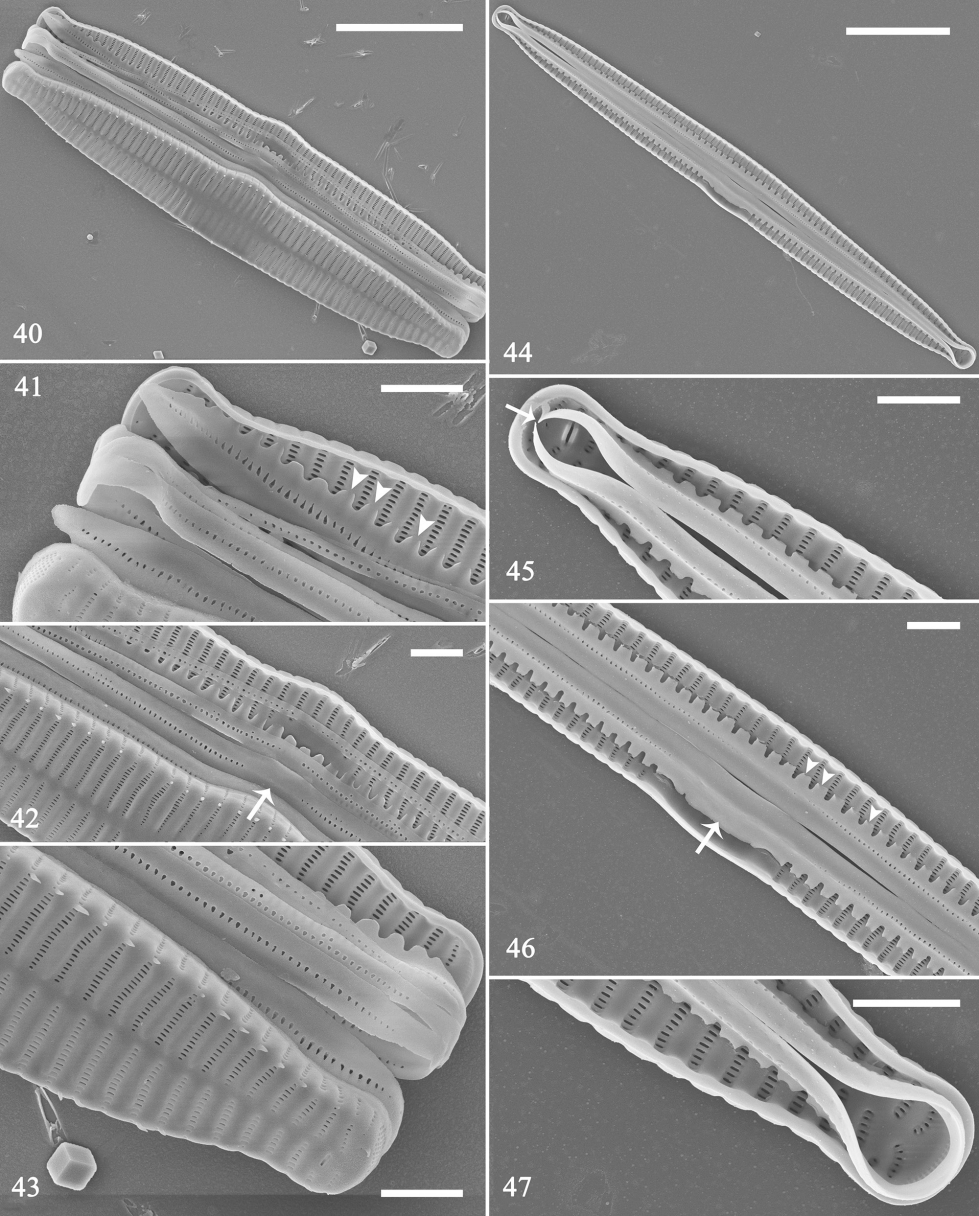
*Hannaea inaequidentata*, SEM**40–43** frustule details showing open girdle bands, note row of poroids interrupted at centre (**42** arrow; also see Fig. 46, arrow) **44** valve with complete valvocopula **45–47** details of Fig. 44 showing open valvocopula (**45** arrow), sawtooth-shaped projections attached to valve, internally visible over each virga (**46** arrows; also see Fig. 41, arrows). Scale bars: 10 μm (**40, 44**), 2 μm (**41–43, 45–47**).

Frustules forming valve face-to-face colonies, via interlocking linking spines (Fig. 35, with three complete frustules, i.e., with epivalve, hypovalve and cingulum; two incomplete; Fig. 36, with two complete frustules). Frustules composed of epivalve, hypovalve, and cingulum of 6–8 open bands. For each vegetative, but not dividing, frustule, a 4:2 configuration of girdle bands visible (Figs 37–39 indicated by 4 and 2), i.e., four girdle bands visible for epivalve, two for hypovalve (Figs 37–39, labelled B1 to B4 and B1 to B2, respectively). For dividing frustule with newly formed hypovalves occurring (Figs 38, 39, two arrows for two new hypovalves), a 4:4 configuration of girdle bands (Figs 38, 39, indicated by 4 and 4), i.e., four girdle bands visible for epivalve and four (with two newly added bands, Figs 38, 39 labelled NB3 and NB4) for hypovalve (Figs 38, 39, upper frustule, labelled B1 to B4 and B1 to NB4). Plaques located at mantle edge, strongly developed, distinct (Fig. 37). Interlocking linking Y-shaped spines (Fig. 37, arrowheads) at valve centre, becoming more acute towards each apex, frustules separate from each other at each apex (Figs 37–39). Girdle bands open, with a row of poroids located at centre line dividing pars interior and exterior (Figs 40–47, Fig. 45, arrow), poroids interrupted at centre (Figs 42, 46, arrow). Valvocopula with crenulated pars interior attaching to valve, internally visible over each virga (Figs 41, 46, arrowheads); copulae with smooth pars interior (Figs 41–43).

#### Normal vegetative valve

Figs 48–69

**Figures 48–59.**
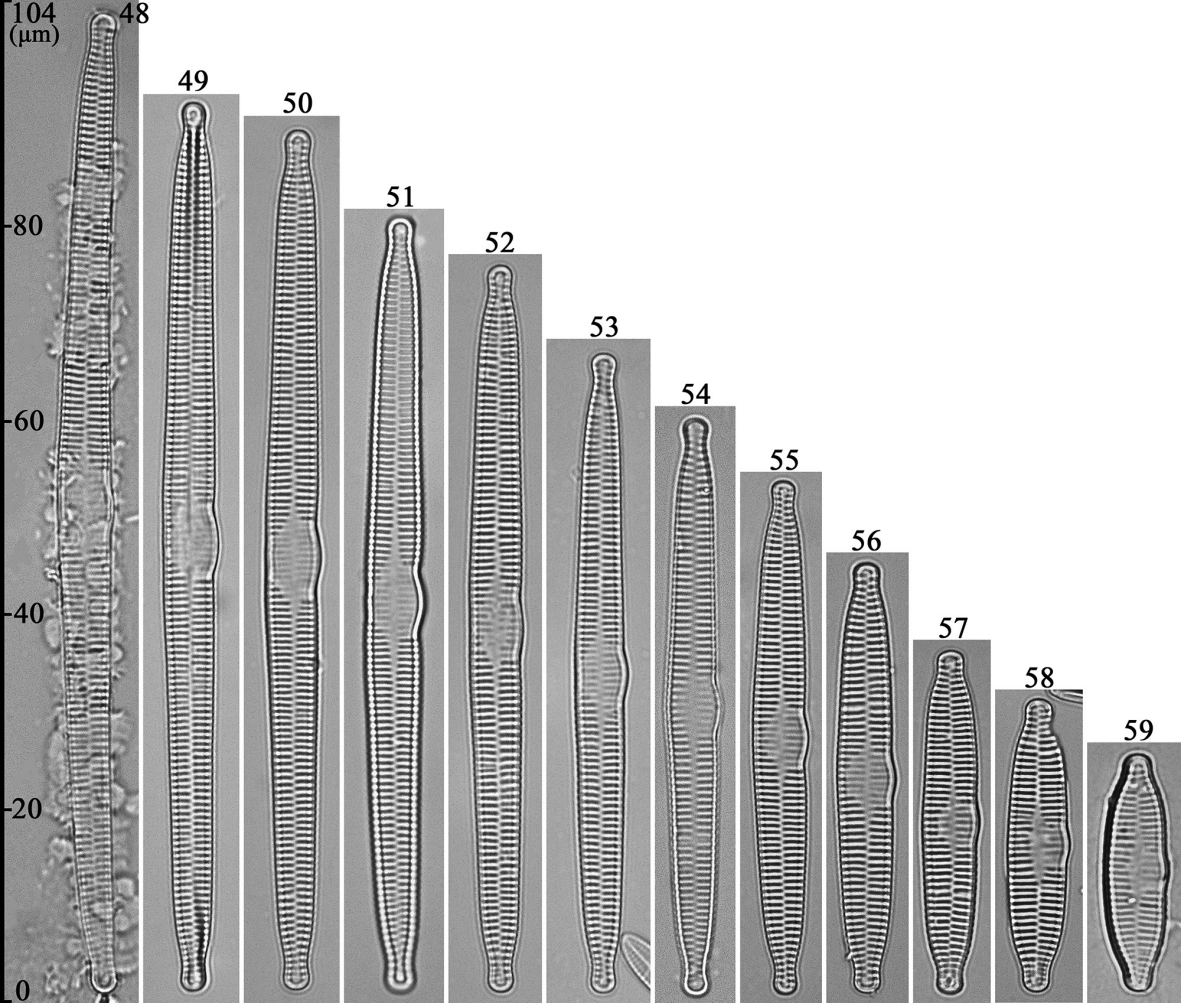
*Hannaea inaequidentata*, normal vegetative valves, LM**48–59** 12 valves showing diminution series, note slightly arcuate, lanceolate valve outlines, and largest valve (**48**) 4× longer than smallest (**59**).

LM: Valves lanceolate, slightly arcuate in larger specimens (Figs 48–55), almost parallel in smaller specimens (Figs 56–59), with capitate to sub-capitate apices. Largest valve (Fig. 48) four times longer than smallest valve (Fig. 59). Valve dimensions (n = 44, Table 1): 24–102 μm long, 5–7 μm wide at the centre. Central area as swelling on ventral side with faint ghost striae. Sternum narrow, almost linear. Striae mostly alternate, parallel, except near each apex where slightly radiate, striae density 14–16 in 10 μm.

**Table 1.** Dimensions of initial valves, pre-normal valves and normal valves in *Hannaea inaequidentata*; values are range (mean ± SD).

	Initial valve length (μm)	Pre-normal valve length (μm)	Normal valve length (μm)
	(n = 7)	(n = 41)	(n = 44)
*H. inaequidentata*	107–139 (122 ± 13)	97–151 (119 ± 12)	24–102 (59 ± 19)

**Figures 60–65.**
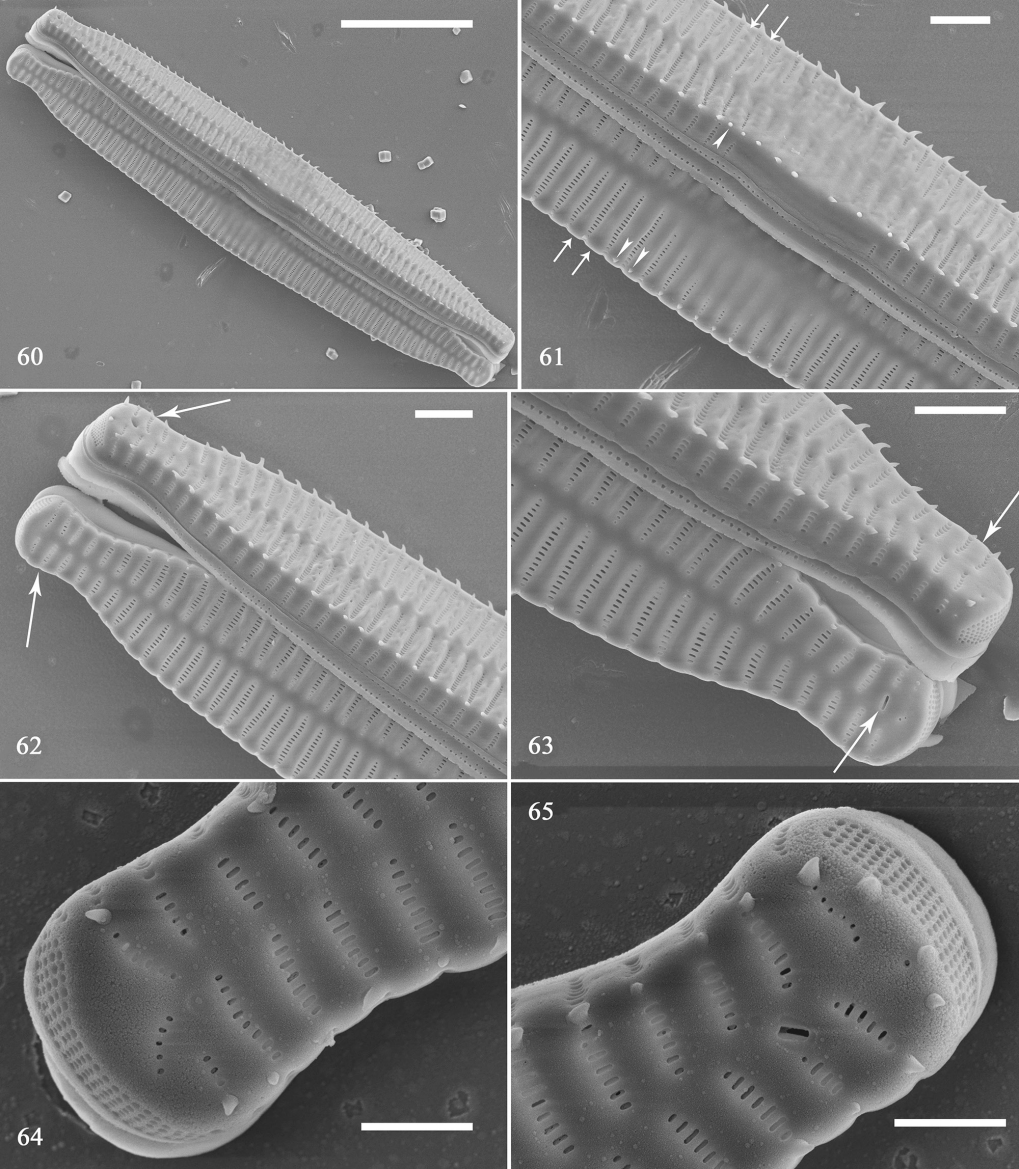
*Hannaea inaequidentata*, normal vegetative valves, external view, SEM**60** displaced frustule **61** detail of Fig. 60, showing well-developed virgae and vimines (arrows), spines mostly located between two adjacent virgae, sometimes situated on virgae (arrowheads) **62, 63** apex details of Fig. 62 showing rimoportula configuration in two valves forming a cell: each cell with two rimoportulae, located diagonally at both apices of each cell (two arrows, respectively) **64, 65** another two apices showing a regular ocellulimbus and areolae occluded internally by hymens. Scale bars: 10 μm (**60**), 2 μm (**61–63**), 1 μm (**64, 65**).

**Figures 66–69.**
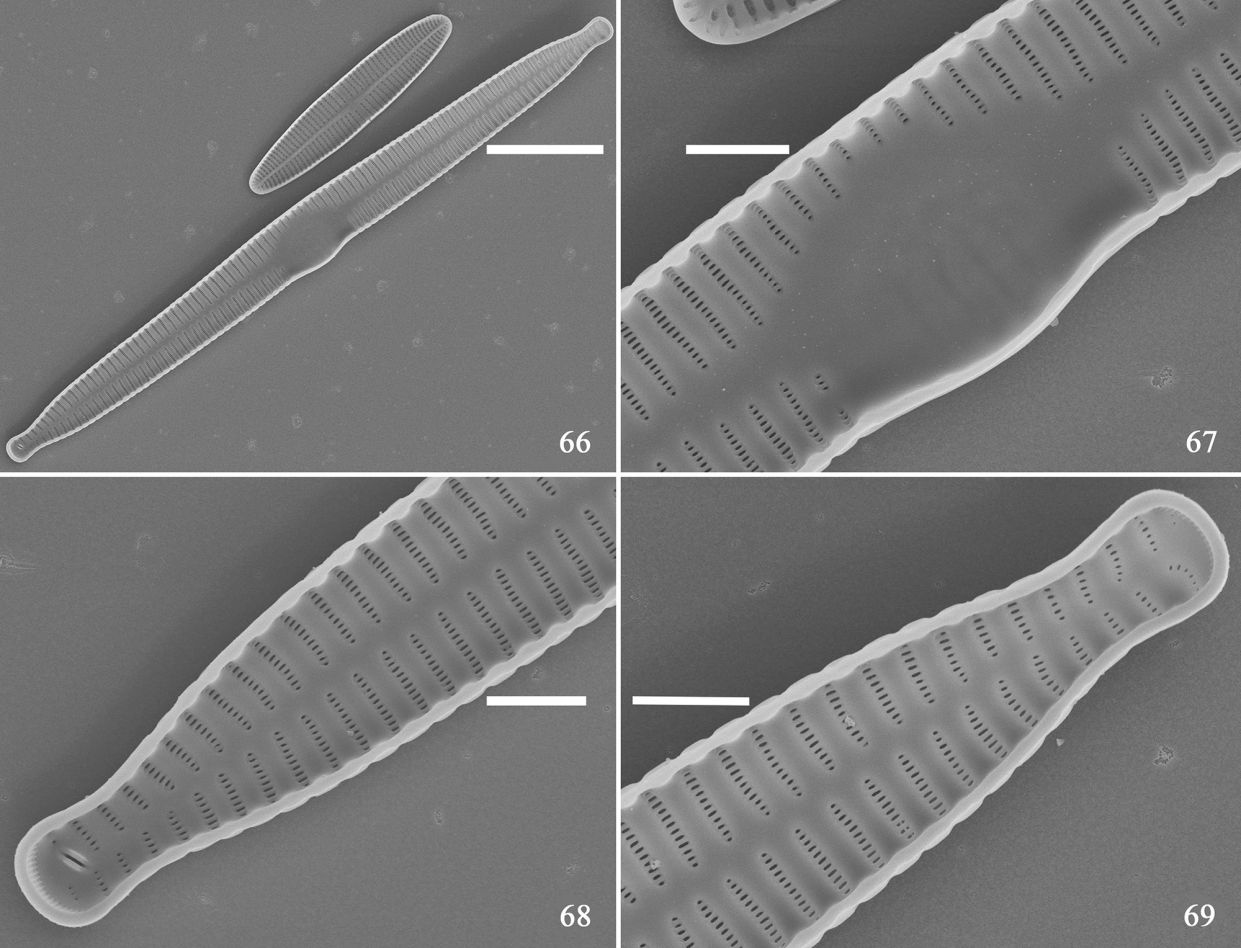
*Hannaea inaequidentata*, normal vegetative valve, internal view, SEM**66** complete valve **67** detail of Fig. 66 showing unilateral swollen middle **68, 69** details of Fig. 66 showing regular sternum and radiating striae near each apex. Scale bars: 10 μm (**66**), 2 μm (**67–69**).

SEM: external view: Virgae raised, vimines depressed on valve surface, spines situated along valve face/mantle junction (Figs 60–65). Spines mostly located between two adjacent virgae within vimines, occasionally on virgae (Fig. 61, arrowheads). Central area with transversely raised virgae, with faint ghost striae (Fig. 61). Each valve bearing one rimoportula at apex, in frustule each pole with one rimoportula (Figs 62, 63, two arrows, respectively). Striae uniseriate, areolae rounded to slit-like, internally occluded by hymens (Figs 64, 65). Ocellulimbus located under valve face, vertical row of poroids composed of ca. 3–7 poroids, and ocellulimbus surface covers valve polar margin (Figs 64, 65). Internal view: valve slightly arched, lanceolate, sternum central, almost straight (Fig. 66). Central area with swollen valve middle margin, virgae raised, vimines depressed, with no apparent buttressing (Figs 66, 67). Rimoportula as paired lips, striae near each apex slightly radiate (Figs 68, 69).

#### Initial frustule

Figs 70, 71, 79–84, 85–90, 91–96

**Figures 70–78.**
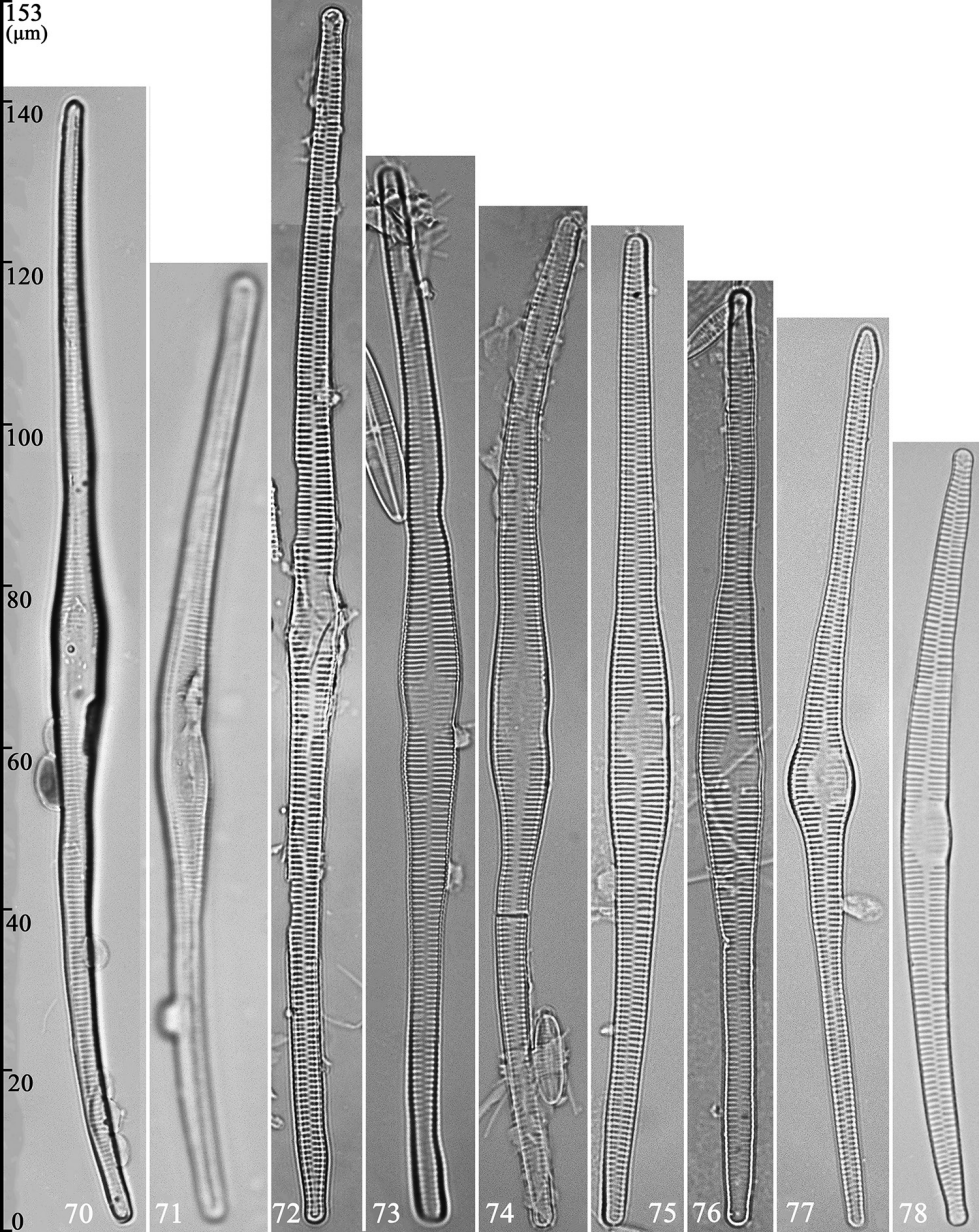
*Hannaea inaequidentata*, initial frustules and pre-normal vegetative valves, LM**70, 71** two initial frustules, note nonexistent (undeveloped) sternum and irregular valve face **72–78** seven pre-normal vegetative valves showing seven irregular valve shapes: almost straight with undulate valve margins (**72**), sigmoid with constricted two middle margins (**73**), double S-shaped with one middle margin constricted (**74**), parallel middle margins with one half of valve straight and the other deflexed (**75**), swollen middle part with almost straight valve (**76**), arcuate with globular middle part (**77**), and nearly normal but distinctly arcuate (**78**).

In LM, the initial frustules have either a curved (Fig. 70) or arcuate (Fig. 71) irregular outline. The sternum is present but not entirely obvious, and the valve face is ill-defined (Figs 70, 71). With SEM, three initial frustules are illustrated to document its fine structure.

**Figures 79–84.**
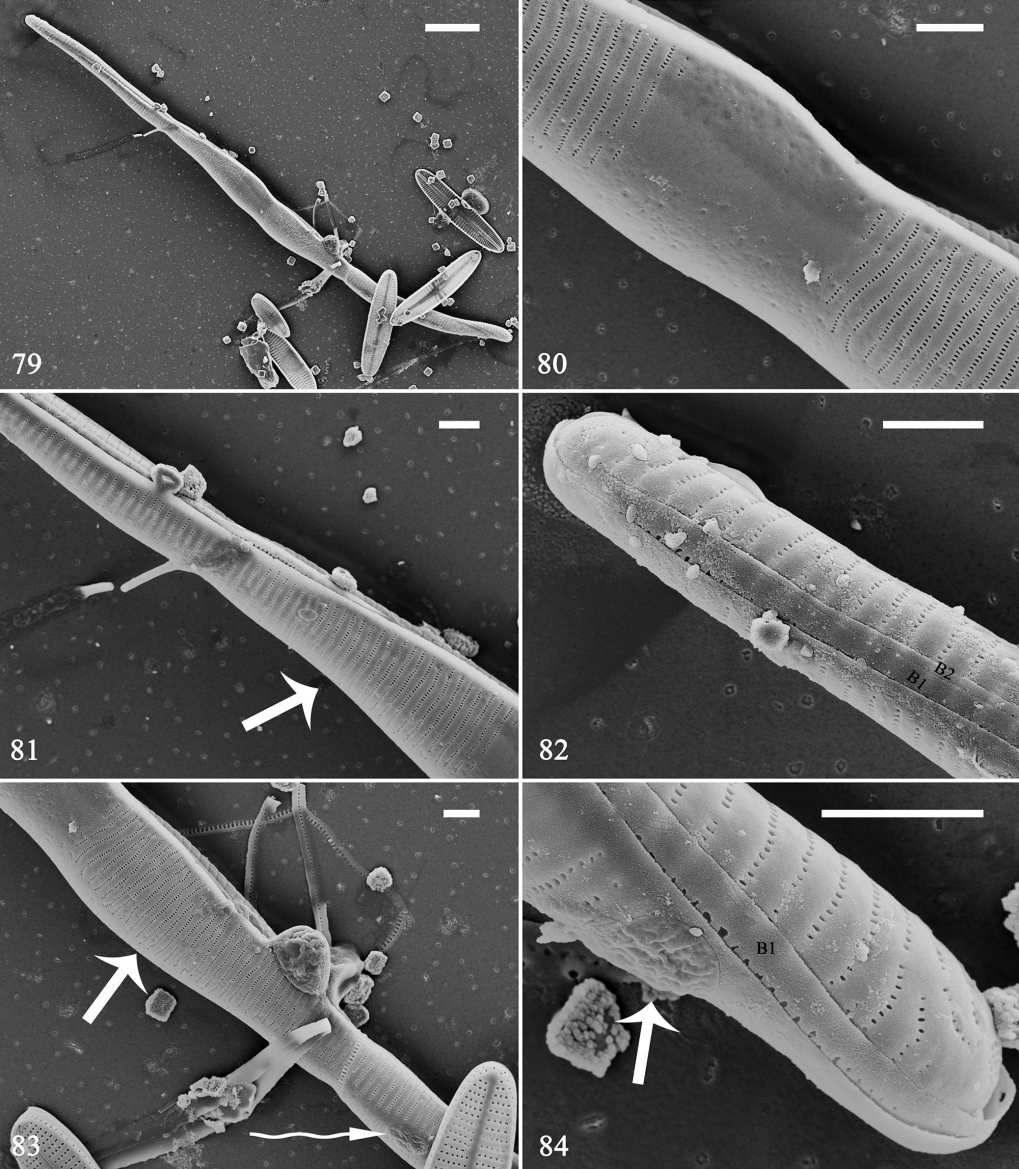
*Hannaea inaequidentata*, an initial frustule, external view, SEM**79** complete initial frustule, note its rounded, cylinder-like, twisted outline **80** middle detail of Fig. 79, showing central area, sternum not developed (i.e. striae continue across valve surface, also see **81, 83**, arrow), longitudinal perizonium wholly covering valve surface, no transverse perizonium bands (also see Figs 85–106) **81** detail of Fig. 79 showing sternum not developed (arrow) **82** apex detail of Fig. 79 showing two girdle bands for this initial frustule, sternum not developed. **83, 84** details of Fig. 79 showing two disc-shaped incunabular scales with cerebral-cortex-like surfaces (**83** curved arrow; **84** arrow). Scale bars: 10 μm (**79**), 2 μm (**80–84**).

The first initial frustule is illustrated in Figs 79–84. It is cylindrical and twisted from pole to pole (Fig. 79). The virgae and vimines are almost flush to each other, with the virgae relatively wide with respect to the vimines (Figs 80–84). The central area is an area completely (or almost) silicified, with no appreciable distinction between virgae and vimines; even ghost striae are not evident, nor is a sternum (Fig. 80; Fig. 81, arrow; Fig. 83, arrow). There are two girdle bands (Figs 82, 84, labelled B1 and B2); the incunabular scales are disc-shaped, slightly dendritic (cf., “dendroid scales (dendroid spine scales)”, Kaczmarska et al. 2013, p. 283; see Fig. 83, curved arrow, Fig. 84, arrow). The perizonium plate cannot be detected because it tightly covers the valve surface.

**Figures 85–90.**
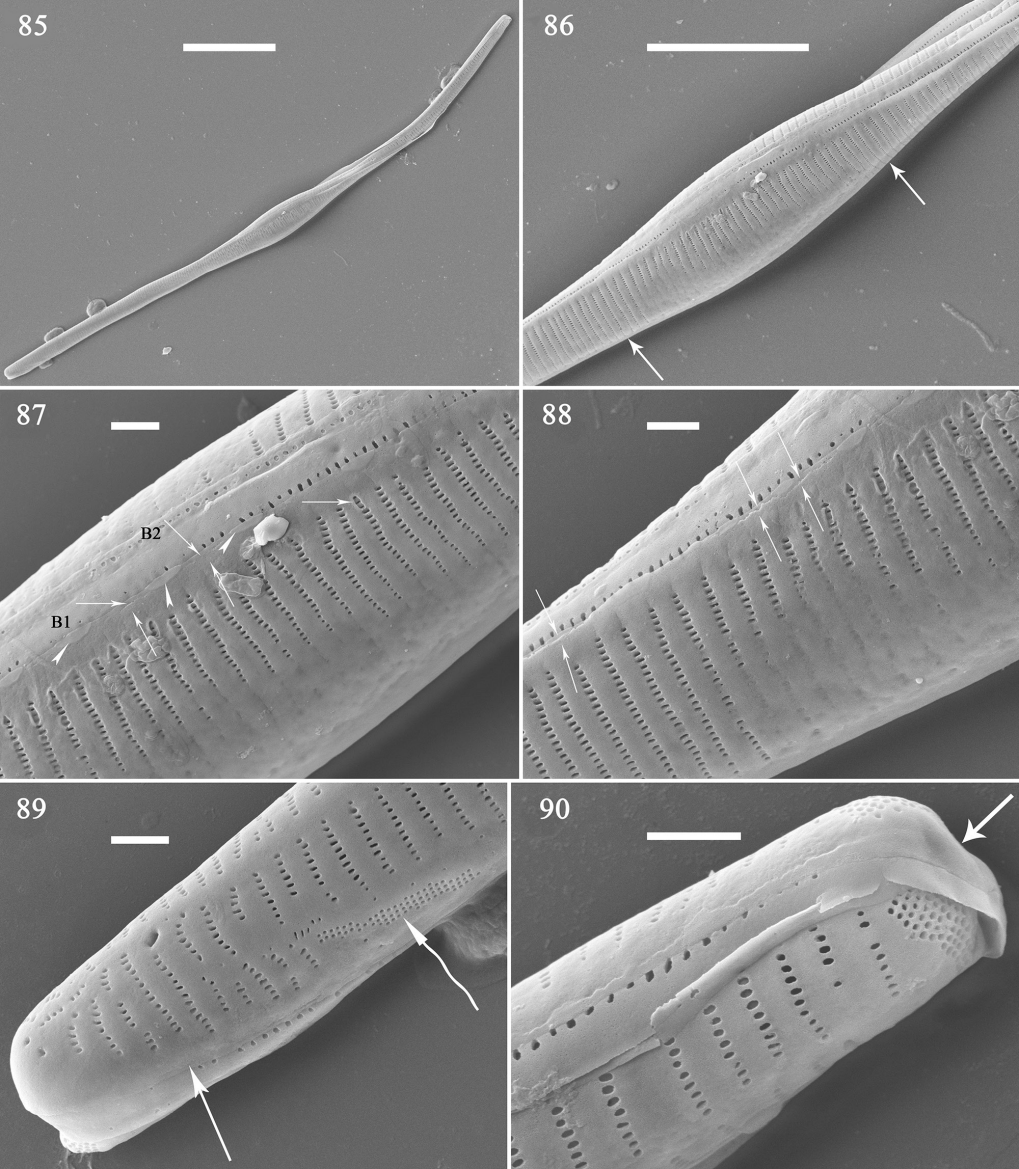
*Hannaea inaequidentata*, an initial frustule, external view, SEM**85** complete initial frustule, note its rounded, cylinder-like, twisted outline **86** middle detail of Fig. 85, showing central area, sternum not developed (two arrows), longitudinal perizonium wholly covering valve surface, no transverse perizonium bands **87** detail of Fig. 85 showing longitudinal perizonium (arrows), plaques (arrowheads), and two girdle bands **88** detail of Fig. 86 showing longitudinal perizonium (arrows) **89** apex detail of Fig. 85 showing longitudinal perizonium (arrow) and irregular ocellulimbus located in valve margin (curved arrow) **90** another apex of Fig. 85, note depressed pole (arrow). Scale bars: 20 μm (**85**), 10 μm (**52**), 1 μm (**86–90**).

The second initial frustule is illustrated in Figs 85–90. It is cylindrical and twisted (Fig. 85). The central area is an area completely (or almost) silicified, with some noticeable distinction between virgae and vimines; ghost striae are evident, but a sternum is not (Fig. 86, two arrows). The longitudinal perizonium plate covers the valve surface, but no transverse perizonium bands were observed (Figs 87–89, arrows). Plaques are present, more spaced out than on the normal vegetative valves (Fig. 87, arrowheads). There are two girdle bands (Fig. 87, labelled B1 to B2). There is a cluster of small poroids on the valve margin giving the appearance of a pore-field or ocellulimbus (Fig. 89, curved arrow); the ocellulimbus occurs at the poles (Fig. 90). The longitudinal perizonium plate approaches a corrugated appearance at one pole (Fig. 90, arrow).

**Figures 91–96.**
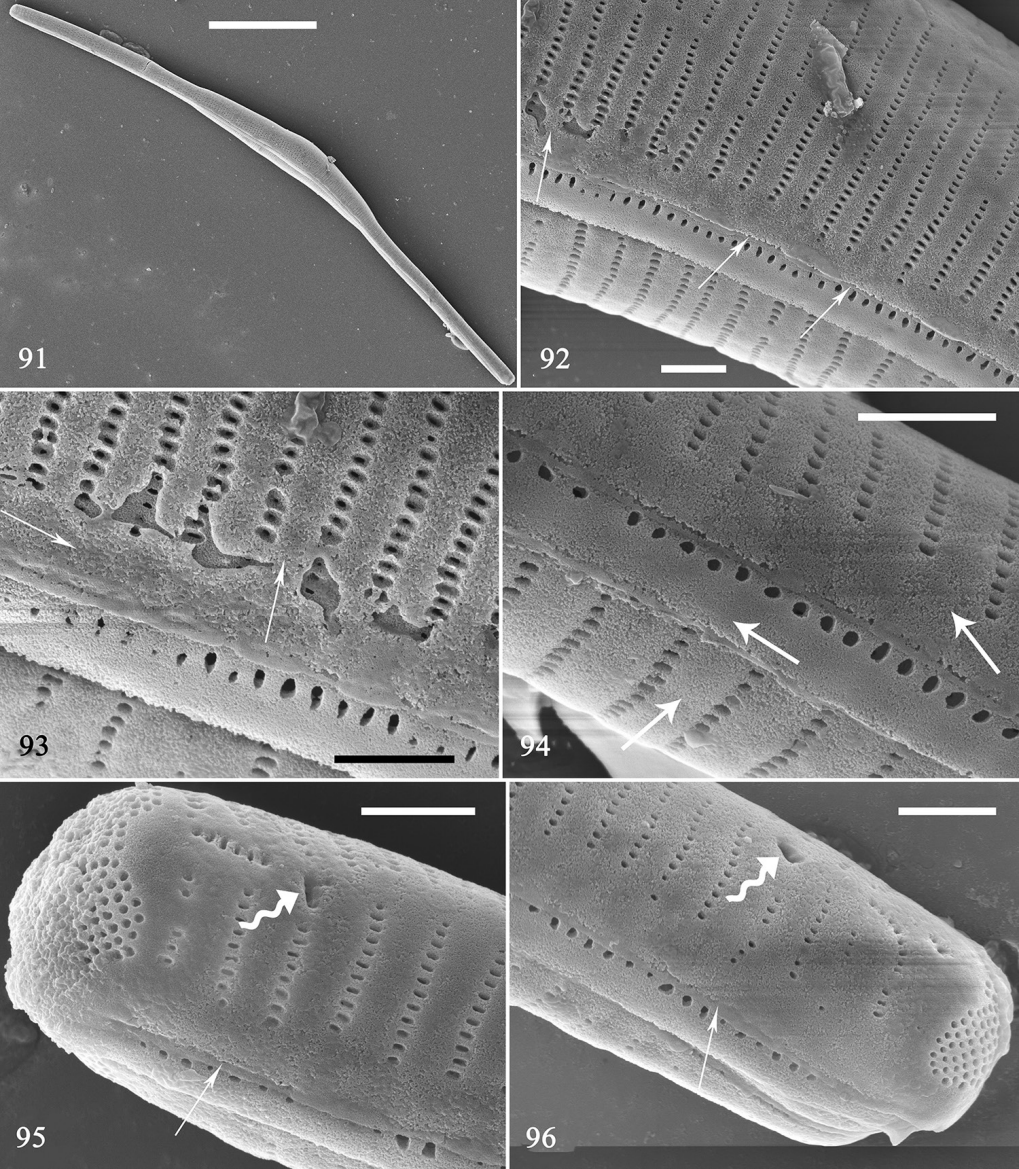
*Hannaea inaequidentata*, an initial frustule, external view, SEM**91** complete initial frustule, note its rounded outline **92** detail of Fig. 90, showing longitudinal perizonium wholly covering valve surface, no transverse perizonium bands (arrows) **93** detail of Fig. 90 showing longitudinal perizonium (arrows) **94** detail of Fig. 91 showing longitudinal perizonium on two valves and one girdle band (arrows) **95, 96** two apex details of Fig. 91 showing longitudinal perizonium (arrows), irregular ocellulimbus, and two rimoportulae per valve (curved arrows). Scale bars: 20 μm (**91**), 1 μm (**92–96**).

The third initial frustule is illustrated in Figs 91–96. It is cylindrical and slightly twisted (Fig. 91). The virgae and vimines are almost flush to each other and the longitudinal perizonium plate can be observed from the centre to the pole (Figs 92–96, arrows): the two valves and one girdle band are all covered by the longitudinal perizonium plate and band (Fig. 92, three arrows). One initial valve has two rimportulae, one at each pole (Figs 95, 96, two curved arrows).

Overall, the three examples illustrate the changes exhibited from a relatively disorganised structure to a more conventional and regular vegetative valve (see Table 2).

**Table 2.** Features of initial cell, pre-normal and normal vegetative in *Hannaea inaequidentata*.

Feature	Initial frustule/valve	Pre-normal vegetative frustule/valve	Normal vegetative frustule/valve
Colony	solitary	solitary	ribbon-like colony
Girdle band number	two	four	not dividing frustule has six, with 4:2 configuration; dividing frustule has eight, with 4:4 configuration
Plaques	present	present	present
Valve outline	cylinder-like, often twisted	irregular (see Figs 72–78)	slightly arcuate, lanceolate
Valve apex	rounded	rounded, cuneate, rostrate, or sub-capitate	capitate to sub-capitate
Sternum	non-existent or lateral sternum	lateral to central sternum	central sternum, i.e. normal, situated on the middle line of valve
Central area	present or ca. as half with short striae to one side	present or ca. as half with short striae to one side	half with short striae in one side
Virga/vimine	virgae and vimines almost flush with each other	vimines slightly lower than virgae	virgae raised, vimines sunken
Linking spines	not present	gradually developed	present and interlocking cells forming ribbon-like colony
Rimoportula number per valve	one, sometimes two	one, sometimes two	one
Ocellulimbus	extending valve face, pervalvar row of poroids not vertical	pervalvar rows of poroids gradually becoming vertical	pervalvar rows of poroids all vertical

**Pre-normal frustule/valve** Figs 72–78, 97–136.

LM: Seven pre-normal vegetative valves are illustrated, each an irregularly shaped valve. Some with almost with parallel margins, one half of the valve linear, the other half deflexed (e.g. Fig. 75), most tapering towards the poles (e.g. Fig. 72); some with an undulating appearance (Figs 74, 76), others slightly sigmoid with constrictions at the central margins (Figs 73, 74). Some have an expanded central area on one side of the valve (e.g. Figs 77, 78), others with the central area across the whole valve from margin to margin (e.g. Figs 73–76), some partially across valve surface (e.g. Fig. 77), and others with central area on one side of the valve but having a distinctly arcuate outline (e.g. Fig. 78).

**Pre-normal frustule with uniparental initial epivalve**

**Figures 97–102.**
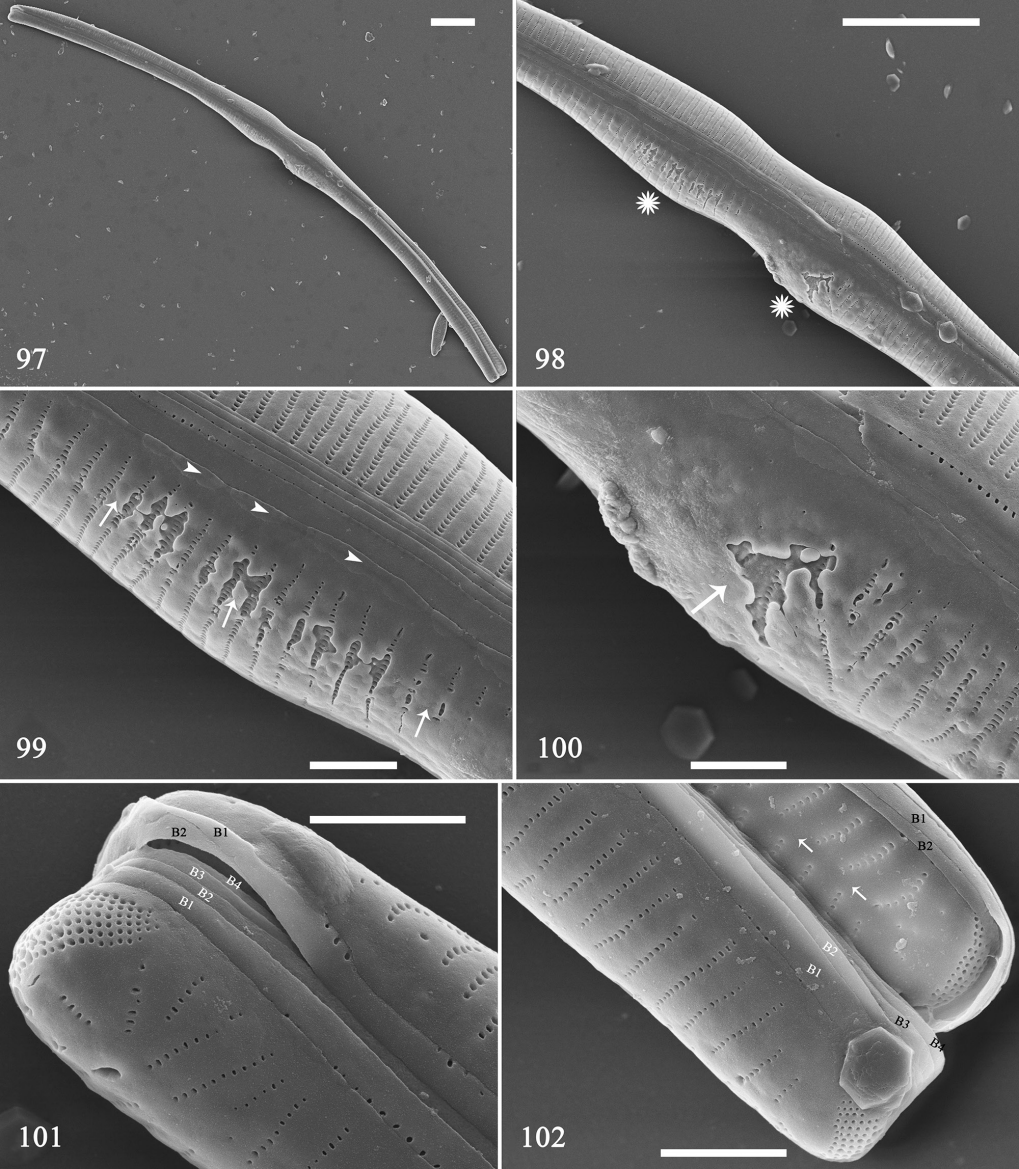
*Hannaea inaequidentata*, dividing half mother frustule, external view, SEM**97** slightly displaced half mother frustule, note its rounded outline **98** middle detail of Fig. 97 showing broken longitudinal perizonium **99, 100** details of Fig. 98 (two asterisks) showing broken longitudinal perizonium (arrows) and distinctive plaques (arrowheads) **101** apex detail of Fig. 97 showing irregular ocellulimbus and 4:2 configuration of girdle bands **102** another apex detail of Fig. 97 showing 4:2 configuration of girdle bands and a new-born hypovalve with regular sternum (two arrows). Scale bars: 10 μm (**97, 98**), 2 μm (**99–102**).

Using SEM, we illustrate two pre-normal frustules with uniparental initial epivalves. The first is illustrated in Figs 97–102. It is cylindrical with a constriction at its centre (Fig. 97). The longitudinal perizonal plate can be observed from centre to pole (Figs 98–102). There are six girdle bands (Figs 101, 102, labelled B1 to B4 and B1 to B2). One new-born hypovalve has a central sternum (Fig. 102, two arrows) and a more conventionally structured ocellulimbus.

**Figures 103–106.**
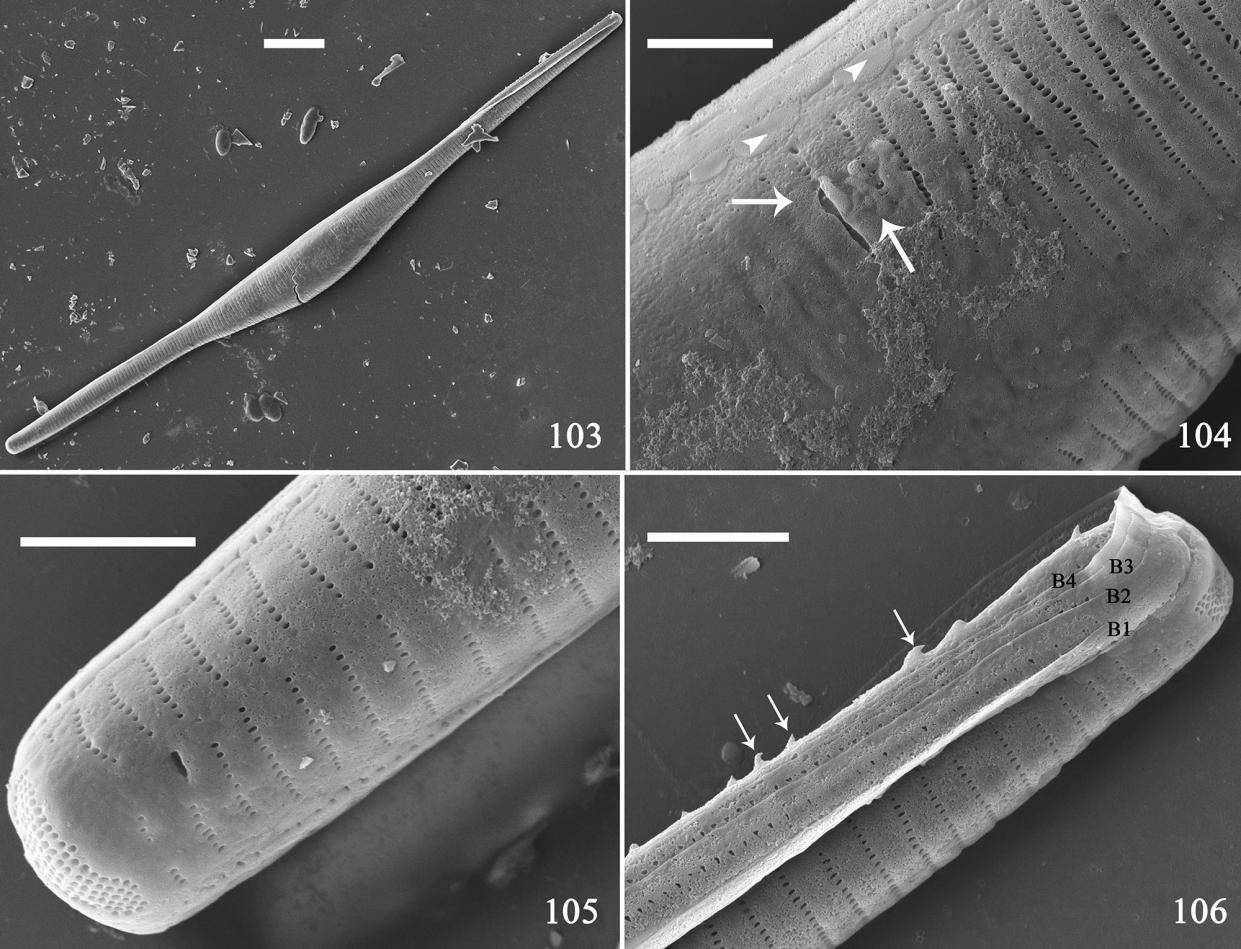
*Hannaea inaequidentata*, half mother frustule, external view, SEM**103** half mother frustule, note its rounded outline **104** middle detail of Fig. 103 showing longitudinal perizonium (arrows) and plaques (arrowheads) **105** apex detail of Fig. 103**106** apex detail of Fig. 103 showing four girdle bands and hypovalve with spines (arrows). Scale bars: 10 μm (**103**), 2 μm (**104–106**).

The second is illustrated in Figs 103–106. It is cylindrical with an expanded central part (Fig. 103). The longitudinal perizonal plate can be observed (Fig. 104, two arrows). The virgae and vimines are almost flush with each other (Figs 104–106). There are four girdle bands (Fig. 106, labelled B1 to B4). The hypovalve has spines (Fig. 106, arrows) indicating that the initial epivalve may have passed a few generations.

**Pre-normal frustule composed of new-born epivalve and hypovalve**

**Figures 107–112.**
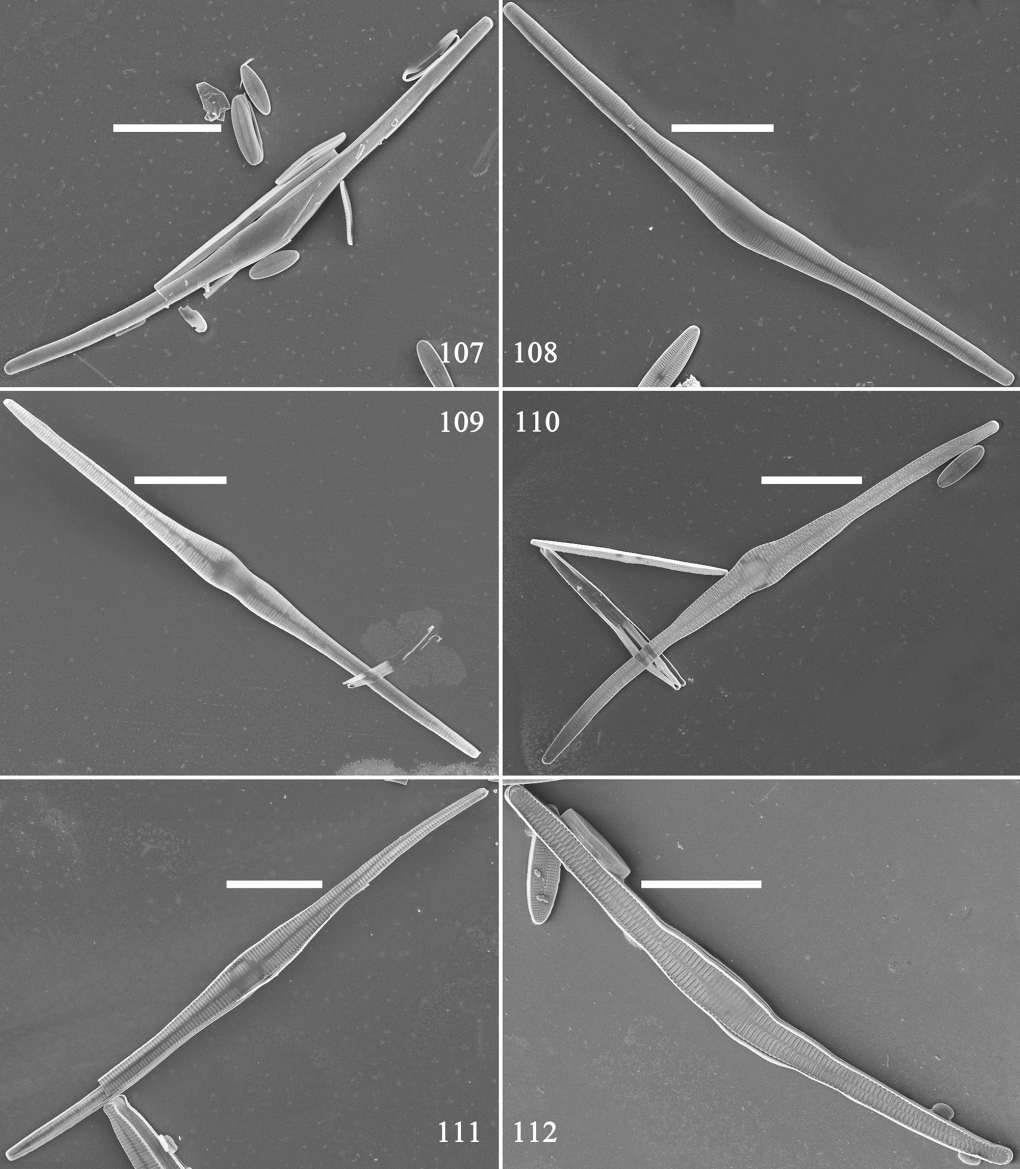
*Hannaea inaequidentata*, pre-normal frustules, external view, SEM**107** frustule with arcuate outline and swollen middle **108** frustule with developed sternum **109** frustule with bi-constricted middle and developed sternum **110** frustule with globular middle and developed sternum **111** twisted frustule with developed sternum **112** frustule with distinct virgae and developed sternum. Scale bars: 20 μm (**107–112**).

**Figures 113–118.**
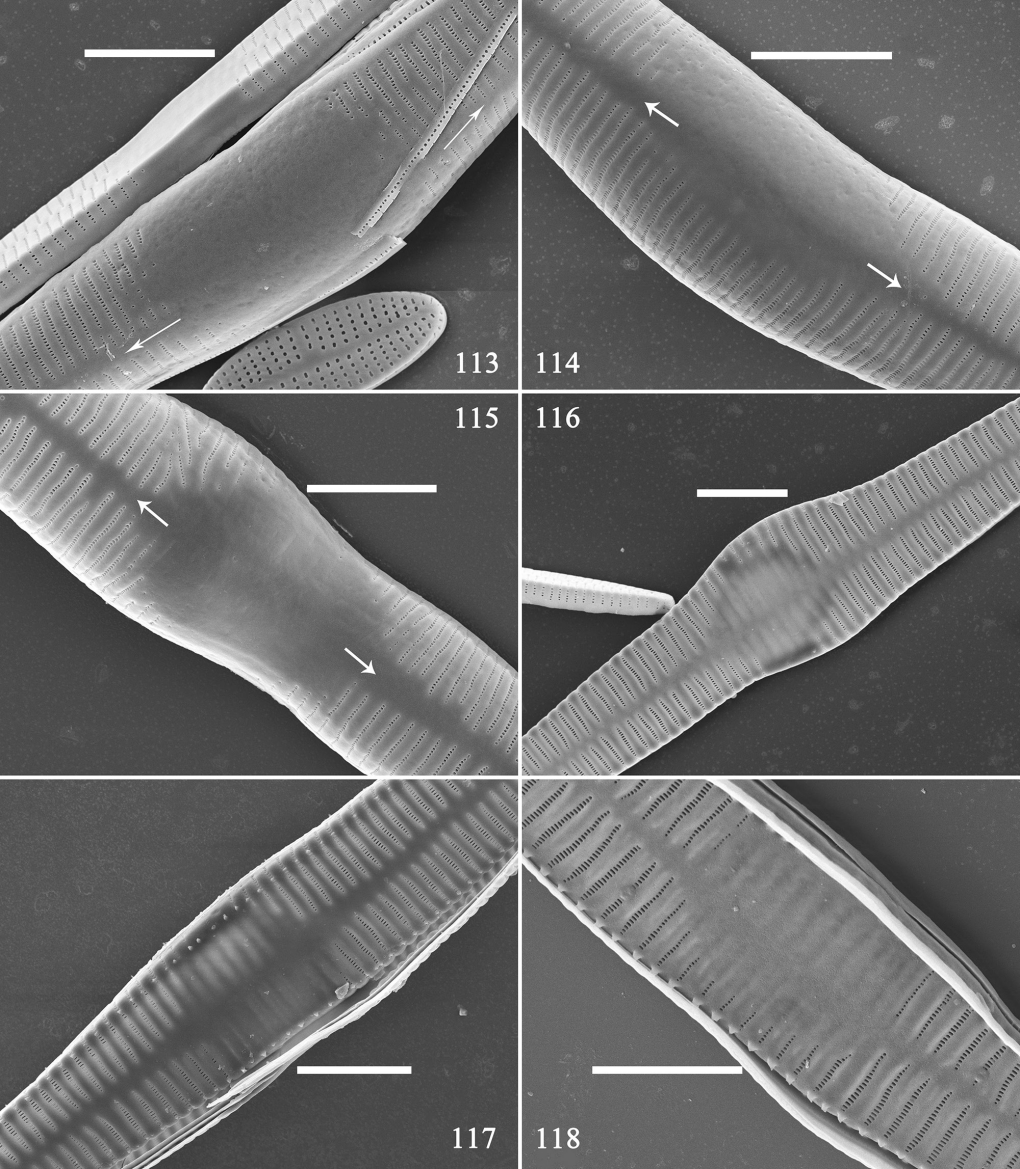
*Hannaea inaequidentata*, middle details of pre-normal cells, external view, SEM**113** middle part of Fig. 107 showing deflexed sternum (two arrows) and central area **114** detail of middle illustrated in Fig. 108 showing sternum (two arrows) and central area **115** detail of middle illustrated in Fig. 109 showing sternum (two arrows) and central area **116** detail of middle part illustrated in Fig. 110 showing developed virgae and vimines **117** detail of middle part illustrated in Fig. 111 showing developed spines **118** detail of middle part illustrated in Fig. 112 showing well-developed virgae, vimines and spines. Scale bars: 5 μm (**113–118**).

**Figures 119–124.**
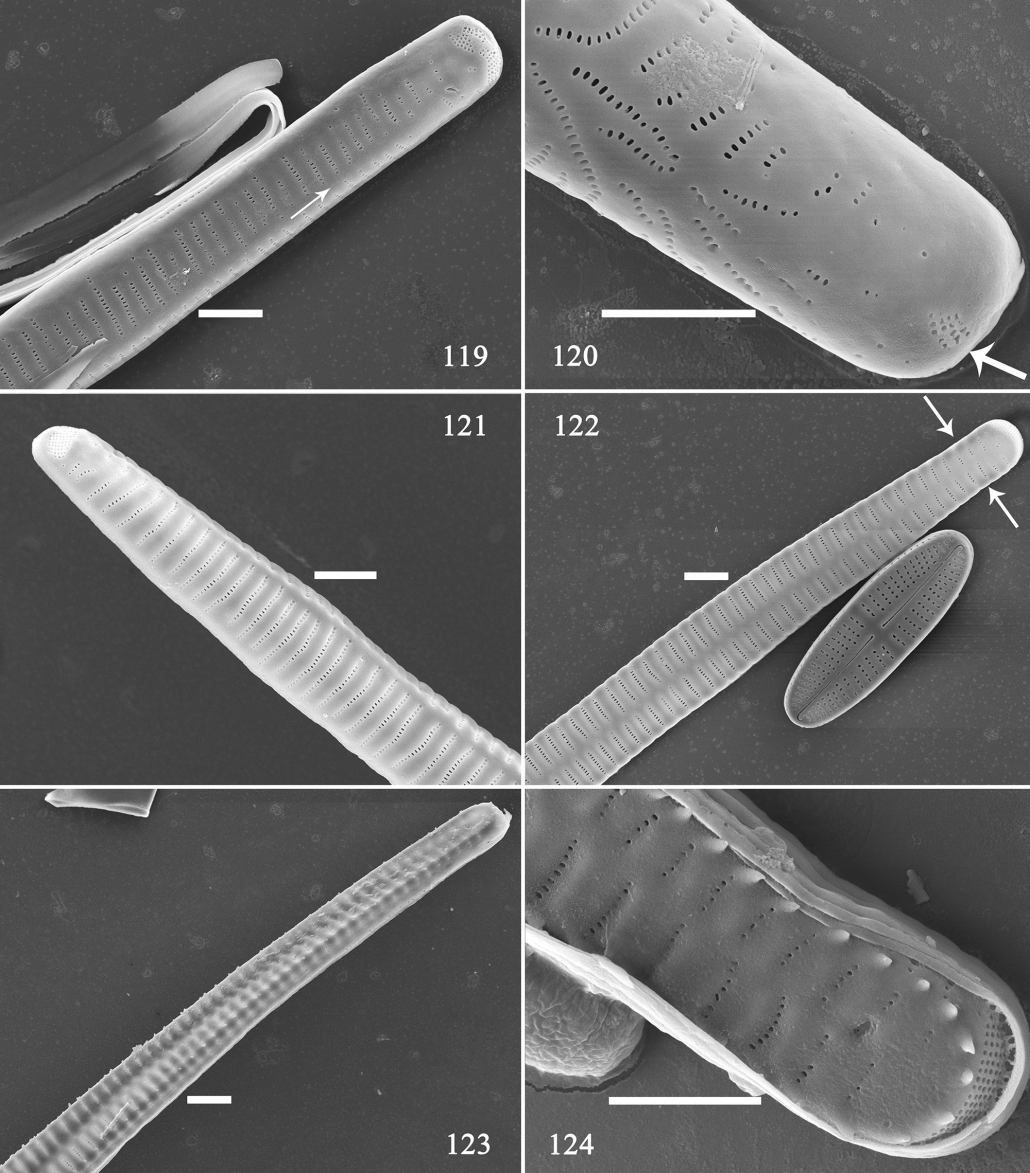
*Hannaea inaequidentata*, apex details of pre-normal cells, external view, SEM**119** apex detail of Fig. 107 showing deflexed sternum (arrow) **120** apex detail of Fig. 108 showing irregular striae and small ocellulimbus (arrow) **121** Apex detail of Fig. 109 showing twisted valve **122** apex detail of Fig. 110 showing almost normal sternum and striae (two arrows) **123** apex detail of Fig. 111 showing twisted valve and not well-developed spines **124** apex detail of Fig. 112 showing well-developed spines and almost normal ocellulimbus. Scale bars: 2 μm (**119–124**).

Using SEM, we illustrate six frustules in external view (Figs 107–112) to document how the pre-normal vegetative frustules gradually develop into normal vegetative frustules (Figs 113–124). The lateral sternum (Figs 107, 113, 119) gradually becomes central sternum (Figs 108–112, 114–118, 120–124). The central area develops from an area without ghost striae (Figs 113–115) and gradually occupies one half of the valve and ghost striae are evident (Figs 116–118). The spines appear forming as outgrowths of a vimine (in most cases) (e.g., Figs 117, 118, 123, 124). At the outset, the virgae and vimines occur on the same plane (Figs 113–115), with the virgae becoming raised away from the vimines (Figs 116–118), and the ocellulimbus gradually become more regular in its structure (Figs 119–124).

**Figures 125–130.**
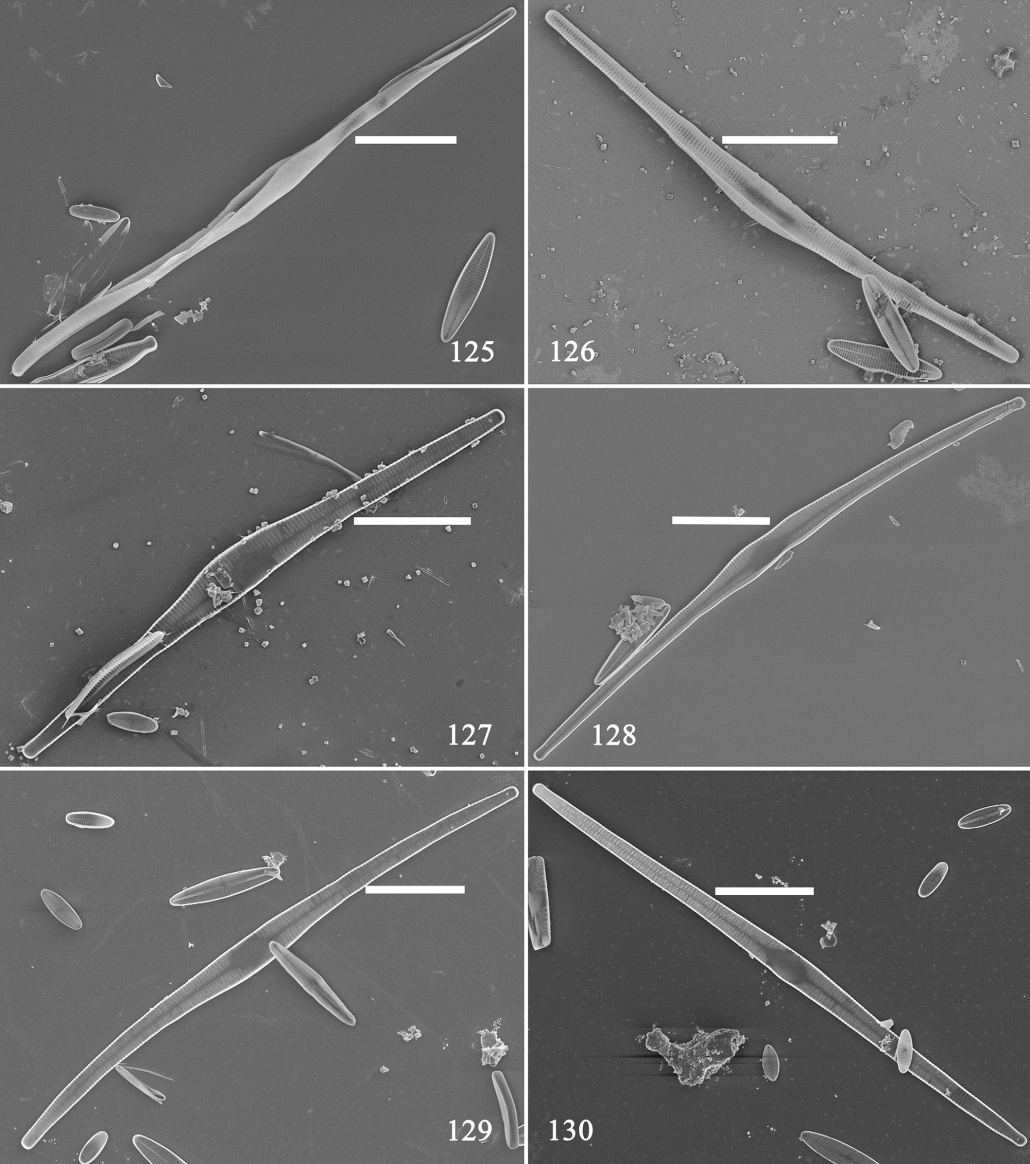
*Hannaea inaequidentata*, pre-normal vegetative valves, internal view, SEM**125** twisted and rounded valve **126** arcuate valve with swollen middle part **127** valve with sternum and swollen middle part **128** valve with bi-constricted middle part and sternum **129** slightly arcuate valve with parallel middle part and sternum **130** nearly normal valve. Scale bars: 20 μm (**125–130**).

**Figures 131–136.**
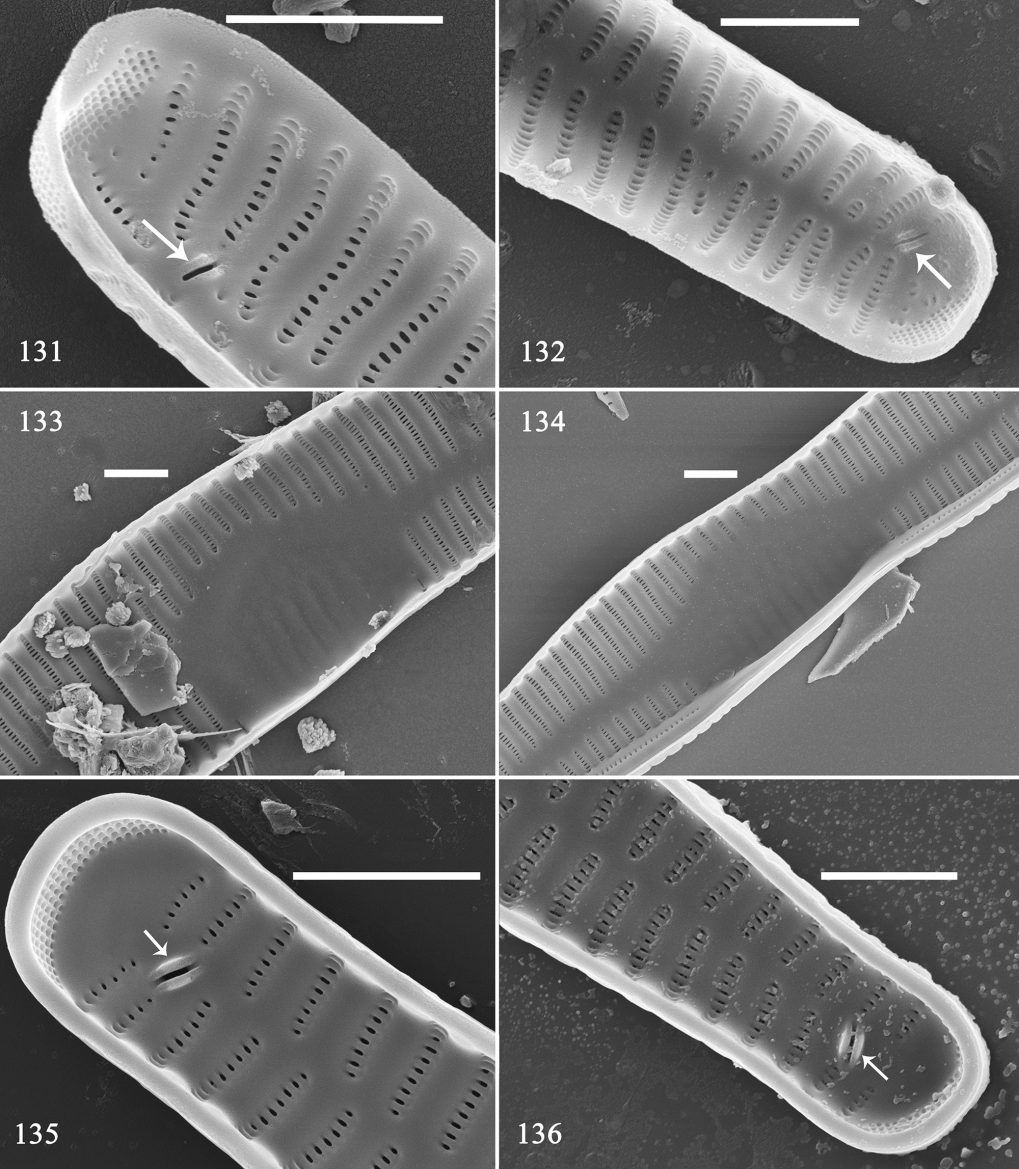
*Hannaea inaequidentata*, details of pre-normal vegetative valves, internal view, SEM**131, 132** two apices of Fig. 126 showing two rimoportulae per valve (two arrows) **133** middle part detail of Fig. 127 showing swollen central area and ghost striae **134** detail of Fig. 128 showing the bi-constricted middle part and ghost striae **135, 136** two apices of Fig. 130 showing two rimoportulae per valve. Scale bar: 2 μm (**131–136**).

Using SEM, we illustrate six pre-normal vegetative valves in internal view (Figs 125–130). These valves have different outlines: a twisted, rounded valve (Fig. 125); an arcuate valve with swollen centre (Fig. 126); a valve with sternum and swollen centre (Fig. 127); a valve with bi-constricted centre area and a central sternum (Fig. 128); a slightly arcuate valve with parallel centre and central sternum (Fig. 129); and a nearly normal valve (Fig. 130). Internally, as noted above for the external view, the virgae and vimines first occur on the same plane, with the virgae becoming raised away from the vimines and the lateral sternum becomes central (Figs 131–136). As with the initial valve, some pre-normal new-born valves also have two rimoportulae per valve (Figs 131, 132, 135, 136, two arrows respectively).

#### Summary of morphological features changing

The morphological features that change during the life circle of *Hannaea inaequidentata* are summarised in Table 2. From initial frustule/valve, via pre-normal vegetative frustule/valve, to normal vegetative frustule/valve, the colony, girdle band numbers, valve outline, valve apex, sternum, central area, virga and vimine, linking spines, rimportula number per valve, and ocellulimbus all gradually become normal (Table 2). The valve plaques are a constant feature, occurring in the initial valve, pre-normal valve, and normal valve.

## Discussion

We noted above that *Hannaea* is usually characterised as having valves “asymmetrical to the apical axis, usually with a small, unornamented tumid area on one side of the center of the valve” (Liu et al. 2019, p. 42) – four groups have been recognised, based on a combination of striae structure and rimoportula number: one group has uniseriate striae and a single rimoportula, another has biseriate striae and two rimoportulae, one at each pole; these two groups are both asymmetrical about the apical axis. The additional two groups are those that have either poorly developed asymmetry to the apical axis or with parallel margins (cf. Liu et al. 2019, p. 42). These latter two groups are those possibly related to *Fragilaria* Lyngbye.

### Initial cell and pre-normal vegetative cell

In ‘araphid’ diatoms there are very few reports of transverse perizonal bands. For example, in *Fragilariforma virescens* (Ralfs) D.M. Williams and Round, Williams noted “no sign of transverse perizonal bands at all” (Williams 2001) and in a species of *Ulnaria* (Kützing) Compère, Williams and Metzeltin noted that the auxospore/initial cells were rather large (in excess of 250 μm), curved along their length, with an irregular basal siliceous layer and the valve outline sometimes interrupted by undulations or a central inflation (Williams and Metzeltin 2004, see also Sato et al. 2004 for further comparisons).

### Structure and ontogeny

Valve changes: This study is primarily based on *Hannaea inaequidentata*, a species with almost parallel valve margins, its overall structure similar to some species currently in *Fragilaria* (as noted first by Cleve 1898; *Fragilaria* as defined by *F. pectinalis* (O.F. Müller) Lyngbye, see Tuji and Williams 2006).

Van de Vijver and Ector have documented the changes in shape of valves in *Ceratoneis amphioxys* such that “a continuum is present from longer valves showing the typical valve morphology of *Hannaea arcus* to shorter valves with the indentations that are typical for Hannaea arcus var. amphioxys” (Van de Vijver and Ector 2020, p. 2, see also Jewson and Bixby 2016). In addition, *Hannaea arcus* and Hannaea arcus var. amphioxys were considered to be synonymous, with Van de Vijver and Ector noting that “Based on the results of the morphological analysis using light microscopy, we propose to treat Hannaea arcus var. amphioxys (Rabenhorst) R.M. Patrick as a heterotypic synonym of *Hannaea arcus* (Ehrenberg) R.M. Patrick” (Van de Vijver and Ector 2020, p. 3); Van de Vijver and Ector record others who have previously expressed the same view: Krammer and Lange-Bertalot (1991, p. 134, as a shape variant, ‘*Umrissvariation*’) and implied in Genkal and Kharitonov (2008, p. 17, pl.1, fig. 8). Many of currently valid taxon names may turn out to be simply stages in individual life-cycles, e.g. Ceratoneis arcus f. trigibba C. Zimmermann (Zimmermann 1915: 36, pl. 4, fig. 10) and the various valves illustrated in Meister (1919) (see Van de Vijver and Ector 2020 for illustration and discussion).

The 1979 terminology paper defined the central area as “an expanded or otherwise distinct portion of the axial area midway along its length” (Ross et al. 1979, p. 518). This definition related more to raphid diatoms than ‘araphid’ diatoms. Bixby et al., in their study of *Hannaea*, suggested some useful additional terms that help describe more accurately the structure of the central area. In valves of *Hannaea superiorensis* Bixby and Edlund (in Bixby et al. 2005, p. 231), internal views shows that the central area is demarcated by a central swollen portion of the valve with an area demarcated by *buttressing* (as in: “buttressed central inflation”, Bixby et al. 2005, p. 235, p. 234, fig. 11). In *Hannaea superiorensis*, the “buttressed central inflation” extends up to the sternum. The buttresses are effectively a pair of heavily silicified virgae situated either side of the demarcated central area enclosing a series of “ghost striae”, the latter being a more heavily silicified set of virgae and vimines but with each visible (Bixby et al. 2005, p. 234, fig. 11). Most species of *Hannaea* have this kind of central area construction, but not all – see *Hannaea tibetiana*, for example, which has a simple plain area demarcated by the virgae and vimines being more silicified in this area (Liu et al. 2019, p. 46, fig. 3; figure 3B is of the ‘plain’ internal view). The buttressing is less obvious in species such as *Hannaea arcus* and *H. inaequidentata*. Here we noted that in the normal vegetative valves, *H. inaequidentata* has a central area on the ventral side of the valve with faint ghost striae, and transversely raised virgae are evident. Further, in the initial cells, the central area appears without any obvious distinction between virgae and vimines, hence ghost striae and the sternum are not evident. In the ‘pre-normal frustule/valve’, the central area varies in shape, from slightly sigmoid, expanded on one side of the valve, extending across the whole valve, margin to margin, often with varying shapes. Finally, the central area occupies one half of the valve and the ghost striae become evident. The implication is that the virgae in the central area being laid down later emerge from the silica basal layer rather than forming first with the vimines and then being filled in. Thus, while the structure called the ‘central area’ is obviously composed of various parts of the valve structure and is now better known, its relevance to taxon relationships remain less than obvious.

### Relationships

At present, it is not clear if *Hannaea*, consisting of all the various groups of species, is monophyletic, in spite of the conclusions offered by Bixby et al. (2005). As we noted above, Bixby et al. (2005) based its monophyly on a combination of the presence of a unilateral inflation, the lack of striae in that inflation, and a valvocopula with an ad valvar crenate margin. None of these characters appear unique (synapomorphic) to *Hannaea* as currently formulated. For example, the asymmetrical valve shape can be found elsewhere in freshwater ‘araphid’ diatoms currently included in *Fragilaria* (e.g., *Fragilaria flexura* Hoff and Lange-Bertalot in Hoff et al. 2011, which is admittedly an unusual species of *Fragilaria*) and, as we noted above, the “small, unornamented tumid area” is also found in a few other species (e.g., *Synedra mazamaensis*Sovereign 1958 (as the current definition of *Synedra* refers to a marine genus, this species clearly does not belong there – it is probably not a species of *Fragilariasensu stricto* either, but that requires further investigation, see Williams & Karthick, In Review, for comments on the name *Synedra*; other species to consider might be *Fragilaria bidens* Heiberg and its relatives). It is also not clear if the four sub-groups noted above are themselves monophyletic or just ‘convenience’ groups to aid identification.

### Final comments

The diversity of species in *Hannaea* is currently recognised by the array of names available, some 30+ for *Ceratoneis arcus* alone, for example. Many of these may turn out to be definable taxa, but others will simply be stages in the individual life cycles, e.g., Ceratoneis arcus f. trigibba (see Van de Vijver and Ector 2020). Schmid (1997) suggested that species in *Hannaea* may simply be teratological forms of *Fragilaria*, in a similar fashion to the tri-radiate cells of *Centronella* M. Voigt. This is certainly a possibility but the work of Van de Vijver and Ector (2020) suggests that while there are shape changes to the valves, they should not be considered teratological forms but natural. That viewpoint is supported here. Nevertheless, it would seem essential at this stage to perform life-cycle studies where possible to ascertain not just how valves form and how exactly valve characters emerge, but to utilise this information to establish evidence for the relationships of taxa at all ranks.
